# Subaqueous free‐standing 3D cell culture system for ultrafast cell compaction, mechano‐inductive immune control, and improving therapeutic angiogenesis

**DOI:** 10.1002/btm2.10438

**Published:** 2022-10-28

**Authors:** Gwang‐Bum Im, Yu‐Jin Kim, Tae Il Lee, Suk Ho Bhang

**Affiliations:** ^1^ School of Chemical Engineering, Sungkyunkwan University Suwon Republic of Korea; ^2^ Department of Materials Science and Engineering Gachon University Seongnam Republic of Korea; ^3^ Present address: Department of Cardiac Surgery, Boston Children's Hospital and Harvard Medical School Boston Massachusetts USA

**Keywords:** acoustic pressure, aggregation, immune modulation, PIEZO1/2, therapeutic efficacy

## Abstract

Conventional 3D cell culture methods require a comprehensive complement in labor‐intensive and time‐consuming processes along with in vivo circumstantial mimicking. Here, we describe a subaqueous free‐standing 3D cell culture (FS) device that can induce the omnidirectional environment and generate ultrafast human adipose‐derived stem cells (hADSCs) that efficiently aggregate with compaction using acoustic pressure. The cell culture conditions were optimized using the FS device and identified the underlying molecular mechanisms. Unique phenomena in cell aggregation have led to extraordinary cellular behavior that can upregulate cell compaction, mechanosensitive immune control, and therapeutic angiogenesis. Therefore, we designated the resulting cell aggregates as “pressuroid.” Notably, external acoustic stimulation produced by the FS device affected the pressuroids. Furthermore, the pressuroids exhibited upregulation in mechanosensitive genes and proteins, PIEZO1/2. CyclinD1 and PCNA, which are strongly associated with cell adhesion and proliferation, were elevated by PIEZO1/2. In addition, we found that pressuroids significantly increase angiogenic paracrine factor secretion, promote cell adhesion molecule expression, and enhance M2 immune modulation of Thp1 cells. Altogether, we have concluded that our pressuroid would suggest a more effective therapy method for future cell therapy than the conventional one.

## 1 INTRODUCTION

Different physical stresses, such as compression, tension, torsion, and shear stress, have been applied to cellular behavior regulation in 2D cell culture systems.^1^, ^2^ Previous research has demonstrated that physical stress‐modulates 2D cell differentiation, proliferation, paracrine factor secretion, and cell survival rate.^3^, ^4^, ^5^ Based on the beneficial changes in cellular behavior induced by the physical stresses applied to the 2D cell culture system,^3^, ^4^, ^5^ the 3D cell culture system has adopted the physical stresses during cell culture. Unlike the 2D cell culture system, the 3D cell culture system applied physical stress primarily for producing cell aggregates^6^ or inducing cell differentiation.^7^ In fact, most studies on 3D cell aggregates have overlooked the critical cellular changes caused by physical stress generated from the 3D cell culture system.

Studies on stem cell spheroids, for instance, have revealed enhanced therapeutic effects and related molecular mechanisms found in cell aggregate. However, there is still a lack of analyses of single cellular events induced by physical stimulation applied from devices to form spheroid.^8^, ^9^ In addition, it is difficult for representative 3D cell culture systems such as the hanging drop method (HD) and forced‐floating method (FFM) to replicate in vivo conditions such as the omnidirectional in vivo microenvironment.^6^, ^8^, ^9^, ^10^, ^11^, ^12^ In particular, the HD method is labor‐intensive, time‐consuming, optimized for short‐period cell culture, and slow for inducing cell compaction, which remained significant issues (S.^13^, ^14^).

Subsequently, using nonadhesive material and a curved bottom design, the FFM method can result in instant forcible cell aggregation through centrifugation, but it can also cause cellular damage during the procedure.^15^ In addition, it is difficult to regulate the size of cell aggregates during cell culture, which is essential for enhancing the therapeutic effect based on oxygen and nutrient supply^6^, ^16^, ^17^ without altering the initial cell culture conditions or cell culture plate design using conventional methods. In light of this, a comprehensive investigation of physical stimuli‐induced cellular behavior change is required for the development of a new efficient 3D cell culture system capable of overcoming the limitations of conventional 3D culture methods.

Among the various physical stimuli applied to 2D cell cultures, previous research has demonstrated that extracellular pressure can upregulate the expression of mechanosensitive genes and proteins. *PIEZO1* and *PIEZO2* are mechanosensitive genes and proteins that regulate cellular functions.^18^, ^19^ Through mechanobiological responses, *PIEZO1* and *PIEZO2* promote cell differentiation or cytoskeletal reconstruction in 2D cultured stem cells.^20^, ^21^, ^22^ However, mechanosensitive genes and proteins in 3D cultured stem cells have rarely been studied. Recent research has demonstrated that mesenchymal stem cell spheroids can be formed through self‐organization via the acoustic levitation technique.^23^ As the study primarily focused on the spheroidal cell aggregate formation and spheroid differentiation, the biological effect on the 3D stem cell spheroid induced by generated physical stimulation, which may influence cellular behavior and therapeutic potential of stem cells, has not been considered. Previous studies employing ultrasound waves also considered the technique to be a type of 3D cell culture system comparable to conventional 3D cell culture systems due to the method's lack of exceptional advantages in cell differentiation.^23^ In addition, detailed changes in the molecular mechanisms that affect gene and protein expressions or comparisons between this method and the conventional 3D cell culture method have not been identified.

This study devised an alternative method for cultivating 3D cell aggregates that circumvents the issues inherent in conventional 3D cell culture techniques. Using acoustic pressure, we developed a subaqueous free‐standing 3D cell culture (FS) device that can generate cell aggregates with ultrafast and efficient compaction (pressuroids). In addition, our FS device can maintain an omnidirectionally homogeneous cellular microenvironment that mimics in vivo conditions more effectively than conventional 3D cell culture techniques. In conventional 3D cell culture systems for generating cell aggregates, the FS device significantly reduced laborious and time‐consuming processes. Moreover, based on paracrine factor secretion, pressuroids demonstrated significantly enhanced M2 polarization and angiogenic effects compared to conventional 3D cell culture systems. Our experimental results revealed the effects of constant pressure‐induced mechanosensitive gene and protein (PIEZO1/2) expressions and subsequent molecular changes in 3D stem cell aggregates, which were rarely observed in previous studies. We examined the cellular mechanisms based on time‐dependent *PIEZO1/2* expressions under constant pressure from the FS device, which increased cellular changes, including cell aggregation, adhesion, M2 polarization, therapeutic angiogenesis, and proliferation.

This study also includes simulation data regarding the cell culture system and environment of the FS device. The discovery of cellular mechanisms and the therapeutic effect of exogenous physical stimulation, such as hydrostatic pressure, using our new subaqueous free‐standing 3D cell culture device may provide opportunities for understanding and developing cell culture systems, that are more similar to the in vivo microenvironment than conventional 3D cell culture systems.

## 2 RESULTS

### 2.1 Acoustophoretic force‐induced mechanism and FS device design

**FIGURE 1:**
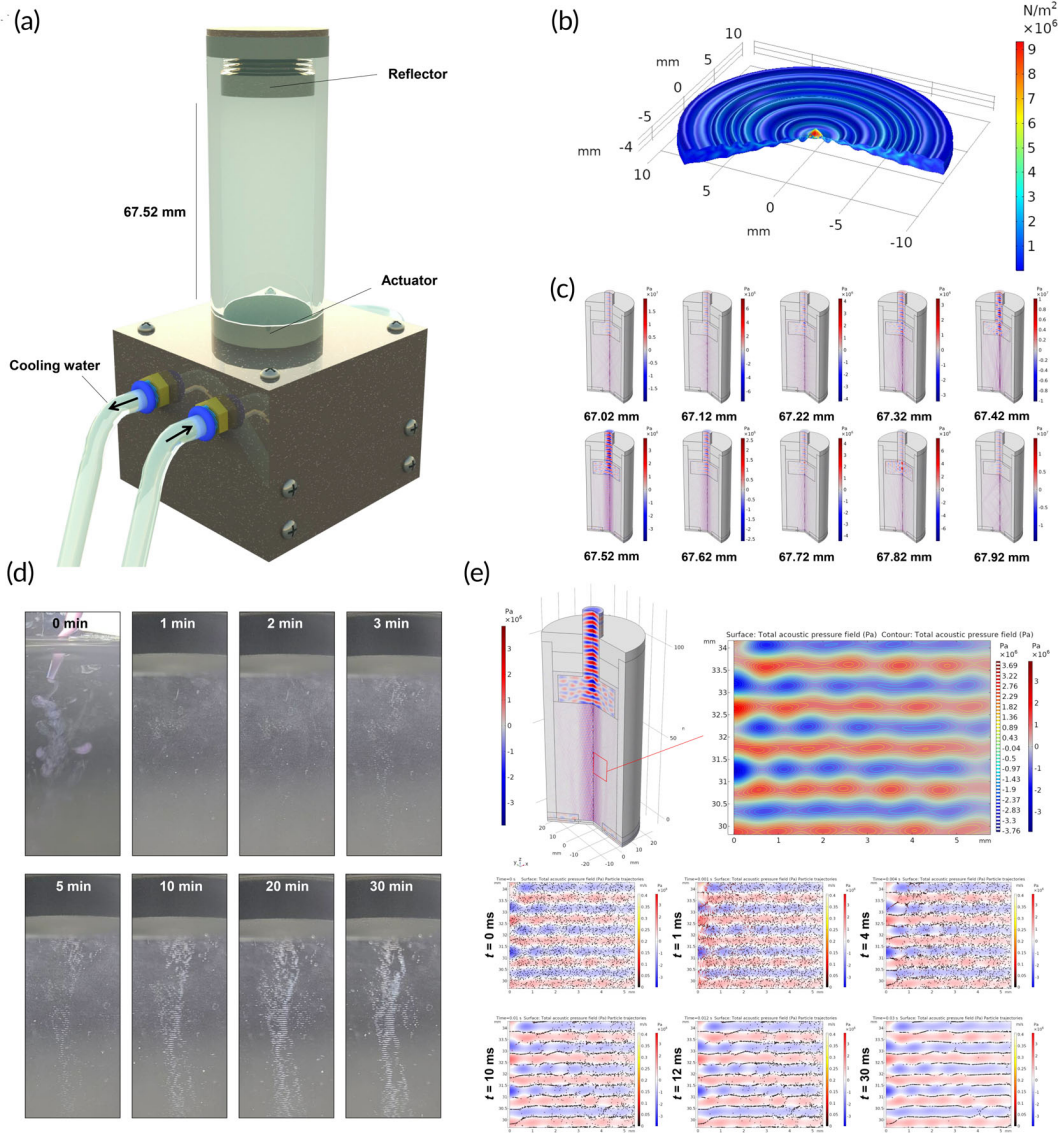
Characterization of FS device. (a) A schematic depicting the components of the FS device. (b) Acceleration magnitude and piezoelectric actuator relative deformation. (c) The acoustic pressure field as a function of height. The maximum pressure field was obtained at 67.52 mm. (d) Representative images demonstrating time‐dependent cell clustering after dispersing hADSCs to the FS device. (e) Acoustic pressure contour plot of a selected region (solid red rectangle) and single‐cell particle tracing results

Subsequently, the FS device was designed to induce subaqueous free‐standing in the cells and cell aggregations using acoustic standing waves in the vertical direction of a cylindrical‐shaped cell culture vessel (Figure 1a). In order to calculate the acoustic standing wave distribution in the cell culture vessel, the pressure generated by the piezoelectric actuator to the water was calculated (Figure 1b). According to the symmetrical design of the actuator, the pressure was greatest at the center and decreased in the radial direction. Figure 1c depicts the calculation results of the acoustic standing wave distribution in the cell culture vessel as a function of the distance of approximately 67 mm between the reflector and the actuator when considering the volume of the medium to be used for cell culture. It was determined that the most stable and uniform standing wave was formed at this distance. Based on the calculation result, the height was set to 67.52 mm by turning the screw holder, and an acoustic standing wave was created in the FS device by applying a voltage to the actuator. Then, the cell suspension was poured into the activated FS device and observed time‐dependent cell aggregation (Figure 1d). The hADSC aggregates were generated within 30 min after activating the FS device.

In order to elucidate the cause of the initial movement of cells by the acoustophoretic force induced by acoustic pressure difference under a stable acoustic standing wave, we constructed a model depicting the situation of cells after pouring a cell suspension. We assumed that a single cell is a spherical particle with a diameter of 30 μm and density of 1050 kg/m^3.^ ^24^, ^25^, ^26^ We placed 4 × 10^5^ particles/device between the actuator and the reflector. The previous calculation's spatial distribution of predefined acoustic standing waves was adopted. Moreover, we calculated the global trajectories of all particles; the trajectories of particles were displayed in a specific region of Figure 1e because the cells were too small compared to the size of the culture vessel. Randomly positioned cells were accelerated and moved to the node regions, leaving their initial positions. Within 0.03 s, the particles stably settled down in node regions. From 0 to 0.03 s, the trajectories of particles are displayed in Figure 1e and Movie S1.

### 2.2 Optimizing cell culture conditions in FS device compared to conventional 3D cell culture methods

**FIGURE 2:**
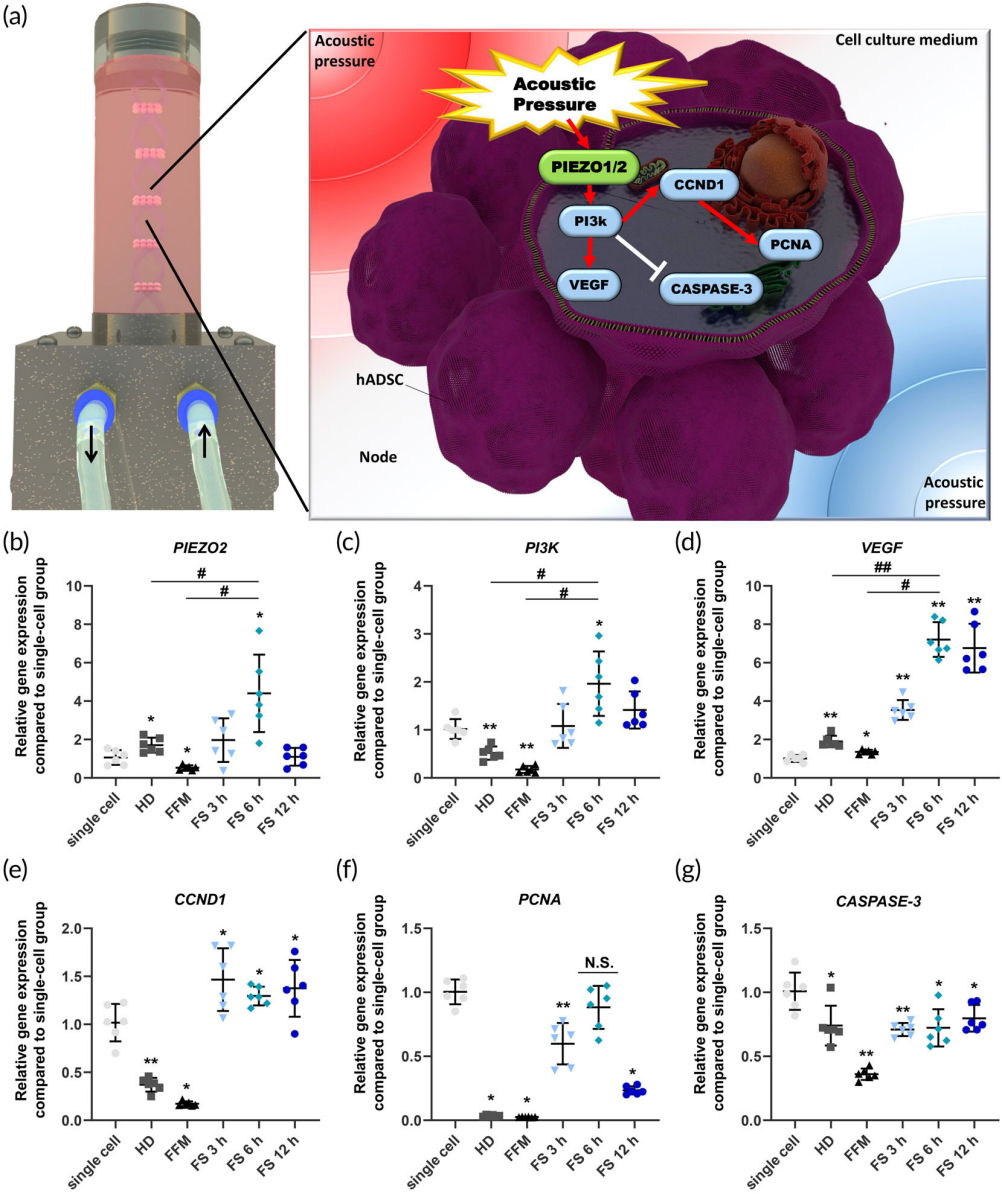
Cellular mechanism analysis of human adipose‐derived stem cells (hADSCs) grown under diverse conditions (single‐cell; FFM, force floating method; FS, free‐standing device method; HD, hanging drop method). (a) Schematic of the cellular mechanism in the FS device that is induced by acoustic pressure. Gene expression of (b) *PIEZO2*, (c) *PI3K*, (d) *VEGF*, (e) *CCND1*, (f) *PCNA*, and (g) *CASPASE‐3* in hADSC under different cell culture conditions (*n* = 6, *p < 0.05 and **p < 0.001 compared with the single‐cell group, #*p* < 0.05 and ##*p* < 0.001 compared to each other, N.S.: not statistically different with single‐cell group)

To determine the effect of acoustic pressure on hADSCs, we compared the gene modification of hADSCs cultured in an FS device to that of cells cultured using single‐cell, HD, and FFM methods (Figures 2 and S1). In fact, acoustic pressure influenced the expression of mechanosensitive ion channel genes, thereby regulating the expression of genes involved in cell proliferation, paracrine factor secretion, and cell death (Figure 2). *PIEZO1* gene expression was significantly upregulated 3 h after hADSCs were cultured in the FS device (FS 3 h) relative to other groups (Figure S1A). The *PIEZO2* gene was elevated in the HD and FS 6 h groups but decreased in the FFM group compared to the single‐cell group (Figure 2b). Compared to the single‐cell group, the FS 6 h and FS 12 h groups displayed the highest phosphoinositide 3‐kinase (*PI3K*) gene expression, which regulates angiogenesis. Figure 2c displays a decrease in *PI3K* gene expression in the HD and FFM groups relative to the FS 6 h and FS 12 h groups.^27^ The expression of the representative angiogenic gene, *VEGF*, was elevated in all groups compared to the single‐cell group (Figure 2d). However, the FS 6 h and FS 12 h groups exhibited the highest *VEGF* expression level compared to the other groups (Figure 2d). Specifically, the FS 6 h group demonstrated significantly higher *PIEZO2*, *PI3K*, and *VEGF* gene expressions than the HD and FFM groups (Figure 2b–d). The expression of the cell cycle regulatory gene *CCND1* was significantly higher in all FS groups (3, 6, and 12 h) compared to HD and FFM groups (Figure 2e). *PCNA* gene expression was downregulated in all groups except the FS 6 h group compared to the single‐cell group (Figure 2f). The *CASPASE‐3* gene was reduced in all groups compared to the single‐cell group (Figure 2g). Based on gene expression results, the FS 6 h group exhibited enhanced proliferation, angiogenic properties, and cell viability compared to other time point results. The FFM group was excluded from the subsequent analysis due to its low gene expression levels for *PIEZO*, *VEGF*, *PI3K*, *CCND1*, and *PCNA*. In addition to the FS 6 h group, the HD group, which had the second‐highest *VEGF* gene expression and is well known for its therapeutic effect on various diseases compared to single cells, was selected for the subsequent analysis.

### 2.3 Comparison of cell aggregation character and re‐adhesion capacity between pressuroid and conventional HD‐derived cell aggregates

First, we studied the size and height of cell aggregates generated using the FS device (6 h, pressuroid). After pouring a suspension of hADSCs into the FS device, the cells moved rapidly toward the acoustic standing wave node and then aggregated. In an acoustic standing wave, the pressure difference is predominant in the vertical direction and approaches zero near the node of the wave. In contrast, due to the symmetry of the acoustic standing wave, the pressure difference along the radial direction is nearly insignificant. Therefore, the vertical growth of cell aggregate is geometrically constrained, whereas the radial growth of cell aggregate is unrestricted.

**FIGURE 3:**
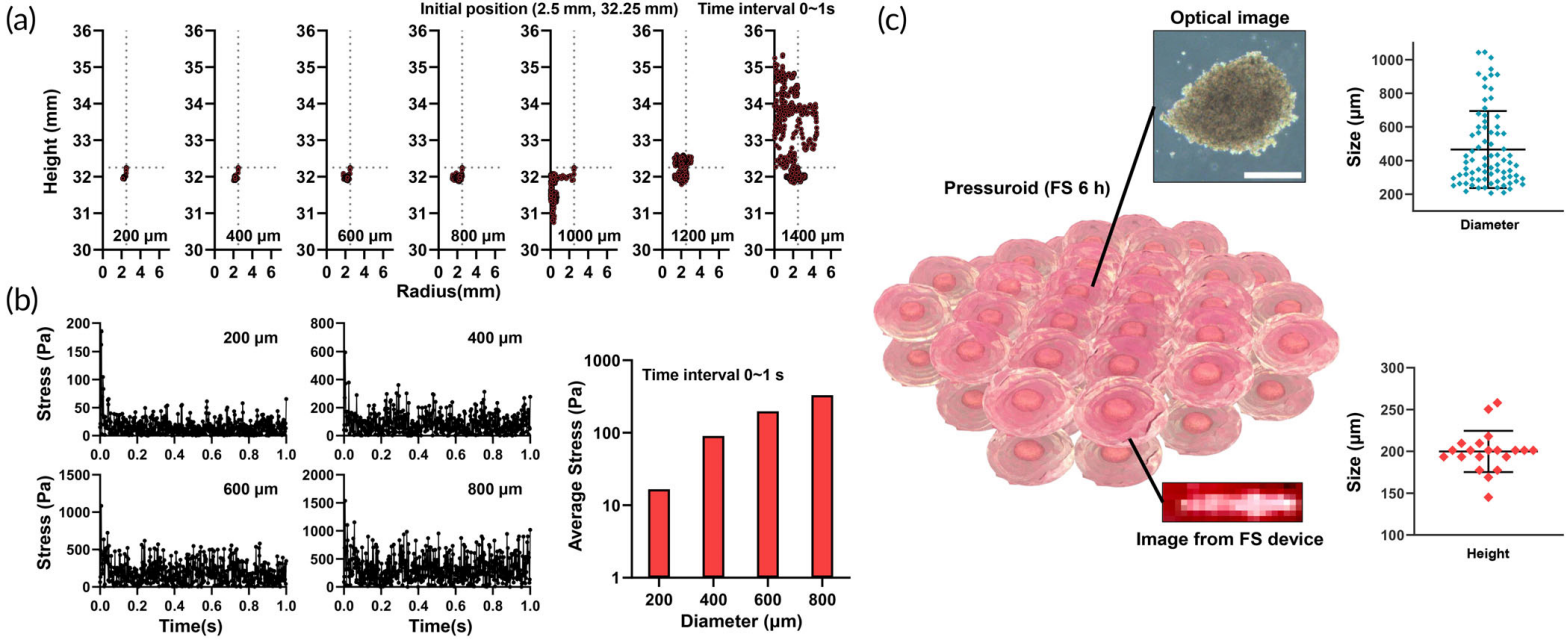
Investigation on cell aggregation of FS 6 h. (a) Single‐particle tracking for investigating the size‐dependent stability of spheroids in an FS device; the red dot indicates the initial position of the particle. The particles size ranges between 200 and 1400 μm. (b) Stress applied to a single‐particle over time, as well as the average stress for 1 s. The scale bar represents 500 μm. (c) The circumference and height of FS 6 h (*n* = 25)

To estimate the effect of the acoustic standing wave on the growth of cell aggregates in a suspended state, we determined the movement of a large particle representing cell aggregates in the acoustic standing wave and the compressive stress applied to the particle surface by water molecules as a function of particle size. We gave the particles a spherical shape and a density of 1050 kg/m^3^. The diameter of the particles ranged between 200 and 1400 μm. All particles were initially positioned at a point (2.5 and 32.25 mm), and their 1‐s trajectories in the acoustic standing wave were computed. The calculation results (Figure 3a) confirmed that particles with a diameter of less than 800 m maintained a relatively stable position near a node, whereas particles with a diameter of more than 1000 μm remained in constant motion. This criterion for suspension stability in the FS device is because the particle size must be less than 937.5 μm for the ultrasound pressures to be nearly balanced (the wavelength of the acoustic wave in this work). Even within the size conditions for stable suspension, the smaller the particle size, the smaller the positional displacement as the magnitude of the acoustophoretic force applied to the particles decreases.

In addition, the magnitude of the compressive stress exerted on each particle within the size range indicating stable suspension was calculated. The stress was calculated every 2 ms for 1 s, and the results are depicted in Figure 3b. Next, the stress exerted on the particles was calculated to fluctuate within a certain range by taking into account the frequency of 1.6 MHz of the acoustic wave and the movement of the particles. As a result, the average value of the stress for 1 s was determined, and it was discovered that the average stress increased as particle size increased. The average diameter of the pressuroids was 470 m ± 220 m, while the mean height was 200 μm ± 24 (Figure 3c).

**FIGURE 4:**
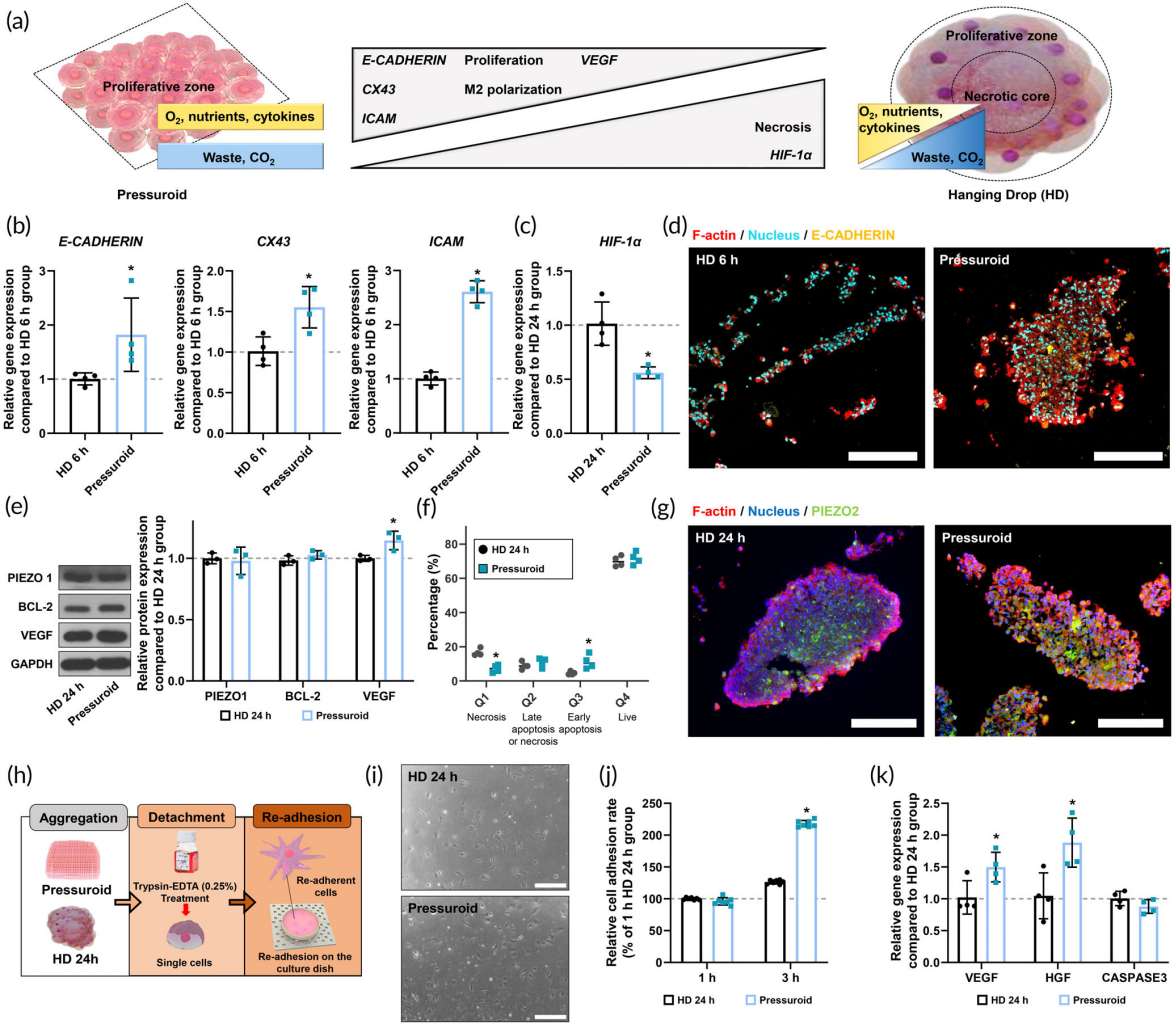
Characterization of FS 6 h compared with HD 6 h and 24 h. (a) Schematic comparing the cellular properties of a pressuroid and a hanging drop. (b) Relative mRNA expression of *E‐CADHERIN*, *CX43*, and *ICAM* in HD 6 h and FS 6 h (*n* = 4, *p < 0.05 compared with HD 6 h group). (c) Relative mRNA expression of *HIF‐1α* in HD 24 h and FS 6 h (*n* = 4, *p < 0.05 compared with HD 24 h group). (d) Immunostaining for E‐cadherin^+^ (yellow), F‐actin (red), and DAPI (blue) in HD 6 h and FS 6 h. Scale bars = 250 μm. (e) Western blot analysis reveals the expression levels of PIEZO1, BCL‐2, and VEGF in HD 24 h and FS 6 h. The graph on the right depicts the quantitative analysis of protein expression as determined by Western blotting (*n* = 3, *p < 0.05 compared with the HD 24 h group). (f) FACS analysis of apoptosis in HD 24 h and FS 6 h cells stained with Annexin V/7‐AAD (*n* = 4, *p < 0.05 compared with the HD 24 h group). (g) Immunostaining for PIEZO^+^ (green), F‐actin (red), and DAPI (blue) in HD 24 h and FS 6 h. Scale bars = 250 μm. (h) Process flow diagram for the re‐adhesion assay of HD 24 h and FS 6 h. (i) Representative optical images of HD 24 h and FS 6 h at 3 h after re‐adhesion. Scale bars represent 250 μm. (j) Relative cell adhesion rate of HD 24 h and FS 6 h at 1 h and 3 h after re‐adhesion (*n* = 6, *p < 0.05 compared with the HD 24 h). (k) Relative mRNA expression of *VEGF*, *HGF*, and *CASPASE‐3* in HD 24 h and FS 6 h after re‐adhesion (*n* = 4, *p < 0.05 compared with the HD 24 h group)

To determine the effect of acoustic pressure on human adipocyte‐derived stem cells (hADSCs), we compared the cellular properties of hADSCs cultured in an FS device to those cultured with HD methods (Figure 4a). Compared to cell aggregates treated with HD for 6 h (HD 6 h group), pressuroid exhibited significantly increased cell‐to‐cell adhesion and gap junction‐related gene expressions (*E‐CADHERIN*, *CX43*, and *ICAM*, Figure 4b).^28^, ^29^, ^30^ Furthermore, pressuroid showed significantly increased *VEGF*, *COX‐2*, and *IL‐10* gene expression compared to HD 6 h group (Figure S2A). In contrast, we found that the expression of *HIF‐1α* in the cell aggregates was significantly lower in the pressuroid group than in the HD 24 h group (Figure 4c). As shown in Figures 4d and S2B, the signals for CX43 and E‐CADHERIN were significantly higher in the pressuroid than in the HD 6 h group, indicating ultrafast cell aggregation. Shear stress broke the cellular structures in HD 6 h and FS 3 h groups, indicating weak aggregation in both groups compared to pressuroids and HD 24 h groups with tight aggregation and intact structures (Figure S2C). Although pressuroids had similar levels of PIEZO1 and BCL‐2 protein expression compared to the cell cluster 24 h after HD (HD 24 h group) (Figure 4e), VEGF protein expression was significantly upregulated in pressuroids. These pressuroids demonstrated a higher population of early apoptosis than the HD 24 h group, whereas the population of necrosis decreased significantly (Figure 4f). As demonstrated in Figure 2, PIEZO2 expression was upregulated in pressuroids relative to the HD 24 h group (Figure 4g). Pressuroid sequentially increased PIEZO2 expression 3 h after increasing PIEZO1 expression (Figures 2 and 4g). After cell aggregation, we separated aggregates into single cells to examine the cell re‐adhesion property and postadhesion cell behavior in order to estimate the cell functions following in vivo cell transplantation at the wound site (Figure 4h). Moreover, 3 h after cell re‐adhesion, pressuroid cells exhibited significantly enhanced cell adhesion properties compared to the HD 24 h group (Figure 4i,j). As *CCND1* is associated with cell adhesion properties, the upregulation of *CCND1* expression in pressuroid increased cell adhesion (Figure 2e). Moreover, *VEGF* and *HGF* gene expressions were elevated in re‐adhesion cells from pressuroids, whereas *CASPASE‐3* gene expression was comparable between the two groups (Figure 4h).

### 2.4 Effects of acoustic stimulation on the immunogenic regulation ability of hADSCs

**FIGURE 5:**
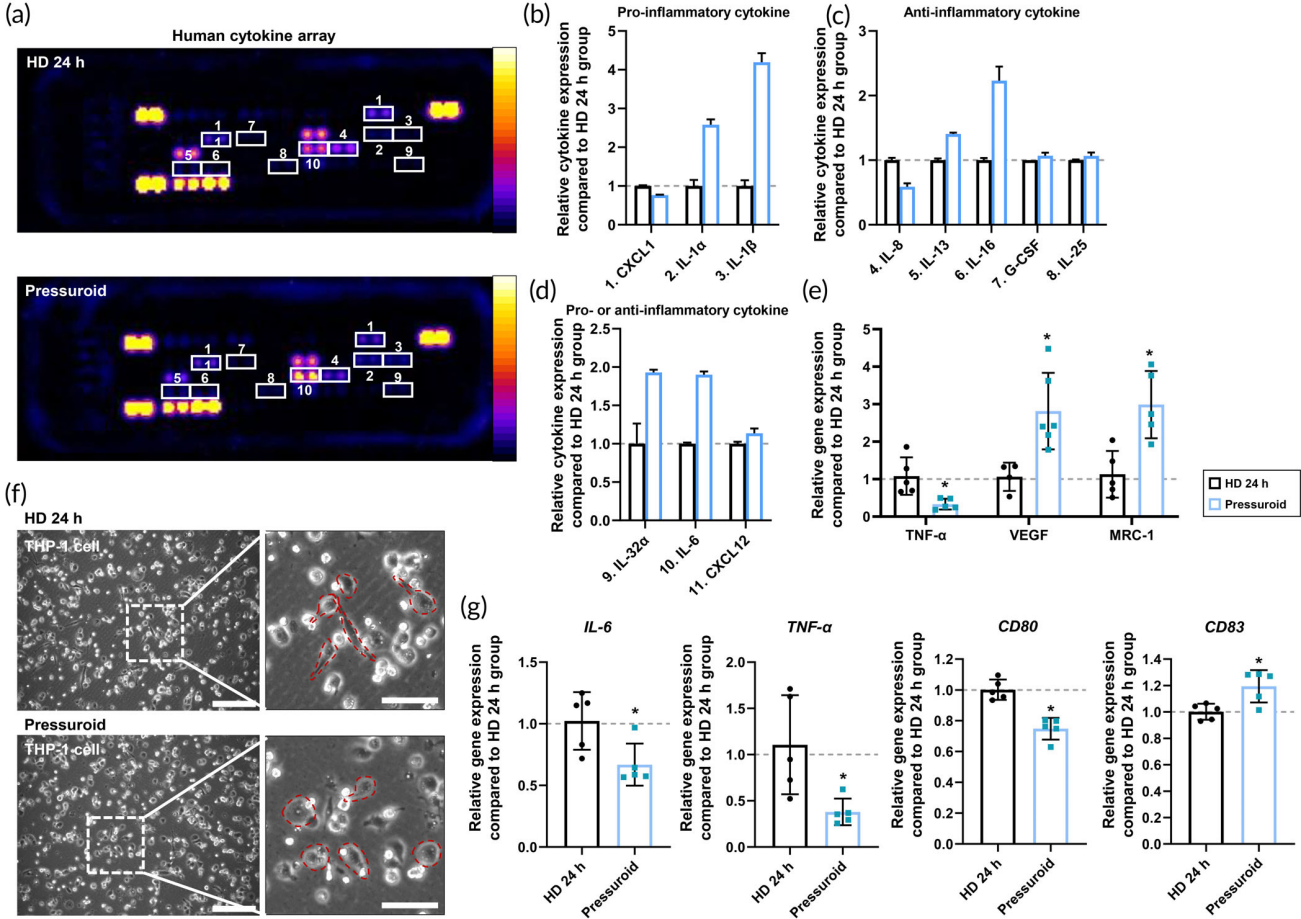
Enhanced immunogenic regulation capacity in FS hADSCs relative to HD 24 h hADSCs. (a–d) Analysis of immunomodulation‐related cytokine expression in FS hADSCs using a human cytokine array. (a) A representative image of the human cytokine array data and (b–d) a comparison of the quantitative differences. (e) Expression of immunomodulation‐related genes (*TNF‐α*, *VEGF*, and *MRC‐1*) in FS hADSCs analyzed with qRT‐PCR. The HD 24 h group served as the control group (*n* = 5, *p < 0.05, compared to HD 24 h group). (f) Morphology of THP‐1‐derived macrophages after treatment with HD or FS hADSCs' condition medium for 24 h. (g) qRT‐PCR analysis of the expression of macrophage marker genes (*IL‐6*, *TNF‐α*, *CD80*, and *CD83*) in THP‐1‐derived macrophages. The HD 24 h group served as the control group (*n* = 5, *p < 0.05, compared to HD 24 h group).

Compared to other cell types, hADSCs do not induce a rapid inflammatory response following transplantation (low immunogenicity).^31^ Additionally, when hADSCs are cultured in 3D aggregates, the ability to secrete anti‐inflammatory cytokines that promote tissue regeneration increases significantly.^32^ Therefore, we examined whether or not the immunogenic responses of the pressuroid (FS 6 h) induced by acoustic stimulation were altered. The cell lysate was initially analyzed using a cytokine antibody array (Figure 5a–d). CXCL1, a proinflammatory factor, was expressed more in the HD group than in the pressuroid group. In contrast, IL‐1α and IL‐1β were significantly elevated in the pressuroid group compared to the HD group (Figure 5b). Both IL‐1α and IL‐1β play crucial roles in cell recruitment and proinflammatory cytokine regulation.^33^ Moreover, when IL‐1 factors secreted by MSCs and hADSCs become autocrine, they stimulate the production of anti‐inflammatory cytokines by the cells themselves.^34^, ^35^ In the pressuroid group, the expression of representative anti‐inflammatory factors (IL‐13, IL‐16, G‐CSF, and IL‐25) was elevated, whereas IL‐8 expression tended to decrease (Figure 5c).^36^, ^37^, ^38^, ^39^ Specifically, the levels of IL‐13 and IL‐16 were significantly elevated. The expression of pro‐ and anti‐inflammatory cytokines IL‐32α and IL‐6 nearly doubled in the pressuroid group relative to the HD group (Figure 5d).^40^, ^41^, ^42^ C‐X‐C motif chemokine ligand 12 (CXCL12), which promotes the migration and recruitment of immune cells in the lesion, was also upregulated (Figure 5d)^43^. Subsequently, quantitative confirmation of changes in the expression of immunomodulatory factors in hADSCs was performed (Figure 5e). Reduced expression of the inflammation‐causing tumor necrosis factor‐*α* (*TNF‐α*) gene.^44^ In contrast, the expression of *VEGF* and mannose receptor c‐type 1 (*MRC‐1*), which decreases inflammation,^45^ was higher in the pressuroid groups than in the HD group. Similar to the results of the previous cytokine antibody array, we confirmed that the gene expression of *IL‐1β*, *IL‐6*, and *CXCL12* was significantly higher in the pressuroid groups than in the HD group (Figure S3). In vitro experiments were used to assess the effects of inflammatory‐related cytokines secreted by pressuroids on immune cells (Figure 5f,g). We investigated the effect of cytokines secreted by pressuroids on the phenotype of macrophages, the innate immune cells at the forefront of the body's immune response.^46^ THP‐1 cells differentiated into the M0 phenotype with PMA were exposed to a conditioned medium from a pressurized culture for 48 h in order to assess macrophage polarization.^47^ THP‐1 cells (M0 phenotype) show a jagged cell morphology prior to differentiation into the M1 phenotype. When cells differentiate toward the M2 phenotype, they assume a round morphology.^48^ In fact, rounder THP‐1 cells were observed in the pressurized group versus the HD group (Figure 5f). In the subsequent analysis of gene expression in THP‐1 cells, the expression of M1 markers *IL‐6*, *TNF‐α*, and *CD80* decreased in the pressuroid group compared to the HD group, whereas the expression of M2 markers *CD83* increased (Figure 5g).

### 2.5 Accelerated wound healing by pressuroid generated from FS device

**FIGURE 6:**
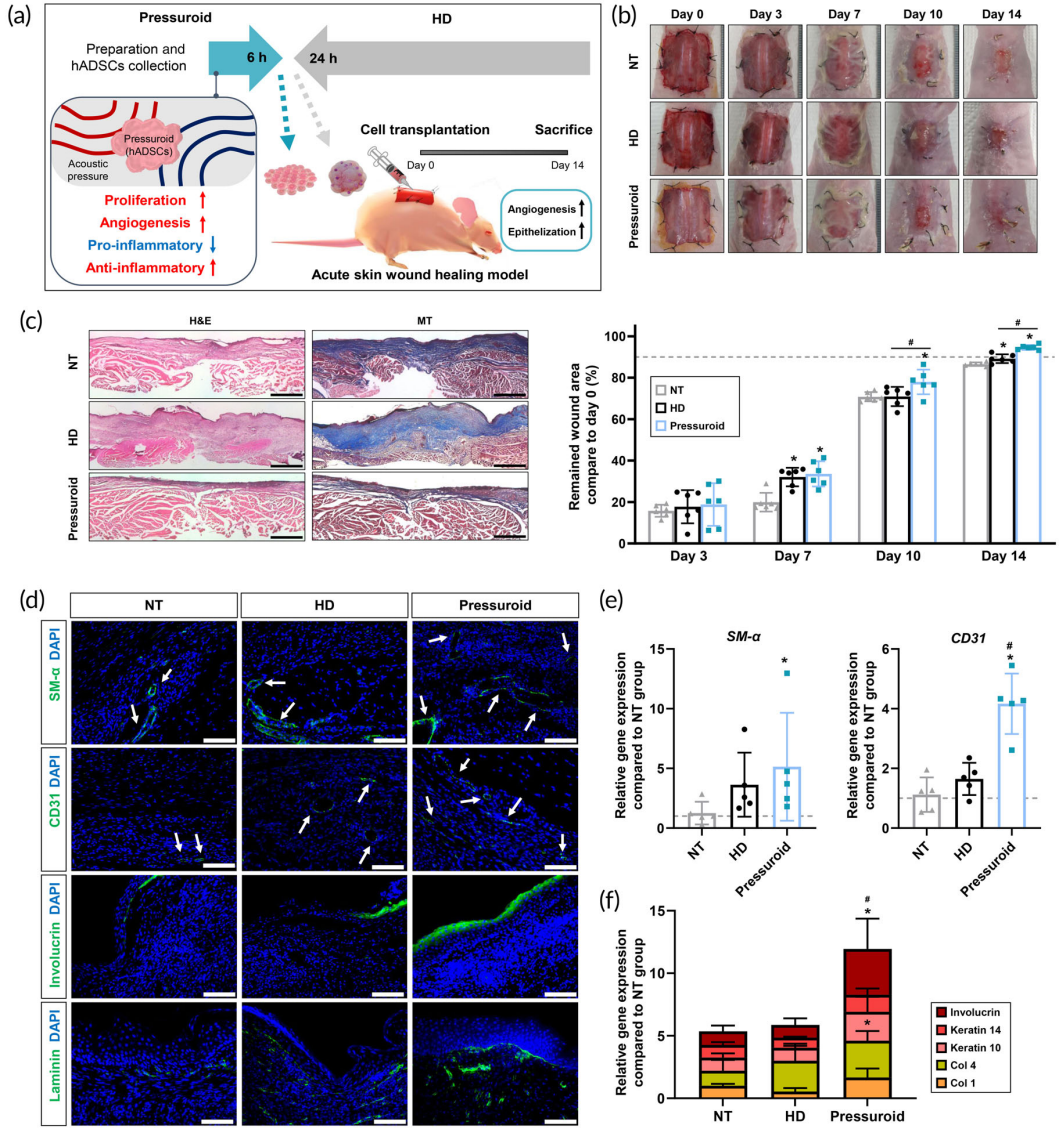
In vivo wound closure induced by FS hADSCs. (a) Schematic of the wound healing model for mouse skin using FS hADSCs. (b) Representative images of the wound on Days 0, 3, 7, 10, and 14 following the various treatments. Quantification of wound closure on Days 3, 7, 10, and 14 following treatment for all groups (*n* = 5, *p < 0.05, compared with that in the NT group, #*p* < 0.05, compared with each group). (c) Representative H&E‐ and MT‐stained images of skin wound model tissue sections 14 days after treatments. (d) Immunohistochemistry for SM‐a (green), CD31 (green), laminin (green), or involucrin (green) with DAPI (blue) in skin wounds 14 days after treatments. (e) Relative expression of SM‐a and CD31 in the wound region 14 days after treatments (*n* = 5, *p < 0.05, compared with that in the NT group; #*p* < 0.05, compared with each group). (f) Relative expression of ECM‐related genes (Involucrin, Keratin 14, Keratin 10, Col 4, and Col 1) in the wound region 14 days after treatments (*n* = 5, *p < 0.05, compared with that in the NT group; #*p* < 0.05, compared with each group)

Subsequently, using a mouse wound healing model, the therapeutic efficacy of the hADSCs in the pressuroid group was evaluated. Following the induction of 2.0 × 2.0 cm^2^, full‐thickness skin defects, mice were treated with 3D hADSCs aggregate transplantation from HD or FS device.^49^ We anticipated that the therapeutic effects of the pressuroid group would be superior to those of the HD group based on in vitro test results (Figure 6a). No treatment (NT), 3D hADSC aggregates collected from HD (0.75 × 10^6^ cells/mouse, HD), and 3D hADSC pressuroids collected from FS device (0.75 × 10^6^ cells/mouse, Pressuroid) were administered to the mice. Figure 6b depicts representative photographs of mice at 0, 3‐, 7‐, 10‐, and 14‐days post‐treatment. Skin wound areas were also measured at each time point. The closure of a wound is expressed as a percentage of the initial wound area. The pressuroid groups exhibited a significantly greater therapeutic effect than the other groups. The HD group demonstrated greater treatment efficacy than the NT group on Day 7, but the difference was insignificant by Day 10 and improved slightly by Day 14. In contrast, the pressuroid group demonstrated greater therapeutic efficacy than the NT group on Day 7, as well as the best results on Days 10 and 14 when compared to other groups. On Day 14, H&E and MT staining confirmed that fibrosis occurred less frequently in the pressuroid group than in other groups (Figure 6c). Blood vessel marker SM‐α and CD31 expressions were evaluated by IHC staining, with the highest SM‐α and CD31 expressions in the pressuroid group versus other groups (Figure 6d).^50^, ^51^ In addition, the gene expression of *SM‐α* and *CD31* was quantified, and the results showed that the pressuroid group had the highest expression levels relative to the other groups. Specifically, the gene expression of *CD31* was dramatically elevated in the pressuroid group compared to the other groups (Figure 6 e). In addition, the expression of skin proteins was confirmed 14 days after treatment. Involucrin is an extracellular matrix (ECM) component that plays a crucial role in the barrier function of the skin. Laminin is an ECM component that makes up the skin's basement membrane.^52^ IHC staining confirmed that the expression of involucrin and laminin in the skin tissue of the pressuroid group was greater than that of the other groups (Figure 6D). The expression of ECM‐related genes, including *involucrin*, *Col 1*, *Col 4*, *Keratin 10*, and *Keratin 14*, in the skin wound area of each group were analyzed.^53^ Col 1 is one of the ECM proteins that make up the dermal interstitial and granulation tissue matrices. Col 4 is an ECM component of the basement membrane matrix.^54^ Increased expression of Keratin 10 inhibits the proliferation of keratinocytes.^55^ In contrast, basal keratinocyte‐expressed Keratin 14 is involved in re‐epithelization during wound healing.^55^, ^56^ In particular, the *involucrin* gene expression was significantly elevated in the pressuroid group compared to other groups. Additionally, the *Col 4* gene expression was significantly elevated in the pressuroid group compared to the NT group (Figure 6f).

## 3 DISCUSSION

Existing 3D cell culture systems struggle with labor‐intensive processes, prolonged cell culture time, and homogenous regulation of cellular function, despite ongoing efforts to develop efficient 3D cell culture systems. In addition, it is difficult to induce rapid cell compaction in a 3D cell aggregate, which is essential for cellular function and viability. This study created a subaqueous free‐standing 3D cell cluster system for ultrafast cell aggregation with compaction, mechano‐inductive M2 polarization, and the enhancement of therapeutic angiogenesis using acoustic pressure control. Previous acoustic levitation technique‐based cell culture methods have yielded intriguing results, such as globular cell aggregation and differentiation without specific gene and protein expression alterations when compared to conventional 2D cell culture methods. In contrast to previous research, we focused on optimizing gene and protein expression to enhance the therapeutic efficacy of 3D stem cell aggregates. In addition to the detailed simulation results of the FS device, several analyses, such as the variation in mechanosensitive molecular mechanisms, immunomodulation properties, and enhanced therapeutic effects in comparison to conventional methods, were investigated. This study demonstrated that pressuroids may be a novel and efficient 3D stem cell aggregate model with enhanced stem cell therapeutic efficacy compared to conventional 3D cell culture techniques.

The FS device was created by generating a single acoustic wave in the cell medium and then spatially constraining it to generate a standing wave to suspend the cells in the culture medium. These acoustic standing waveforms create a water pressure barrier in the medium, allowing cells to hover in the middle of the medium without a solid‐state supporter, with the expected result of rapidly forming cell aggregates by collecting individual cells at node points. The calculation results in Figure 1e explain the rate at which individual cells form a cluster in an acoustic standing wave. The contours represent locations with identical pressure values, and the direction of acoustic pressure difference is perpendicular to each contour line. In the white‐colored node regions of Figure 1e, the pressure difference becomes zero. The acoustophoretic force accelerates the particles in the direction of the pressure difference, and the force becomes zero in the node regions. Within a few microseconds, cells aggregated toward node points in the calculation depicted in Figure 1e, and as shown in Figure 1d, it was confirmed that cell clusters of a visible size were formed within minutes.

As expected, the acoustic pressure in the FS device significantly shortened the cell culture period and labor‐intensive process, which have been identified as issues with conventional 3D cell culture methods.^57^, ^58^ In addition, we analyzed the effect of acoustic pressure in FS devices on stem cell behavior, including proliferation, compaction, cell cycle, cell death, and therapeutic potential after PIEZO1/2 upregulation.^59^, ^60^, ^61^ The relative mRNA expression of *PCNA*, *E*‐*CADHERIN*, *CX43*, and *ICAM* confirmed that by optimizing 3D stem cell aggregate culture conditions with the FS device, the pressuroid rapidly generated enhanced cell proliferation and spheroid compaction. Previously, upregulation of PCNA, which functioned as a processivity factor for DNA polymerase δ, increased cell proliferation and enhanced wound healing.^62^ E‐cadherin, Cx43, and ICAM, among other CAMs, enhance the self‐renewal and multipotency of stem cells.^63^ E‐cadherin is a crucial mediator for intrinsic cell–cell contacts during cell aggregation among these CAMs.^64^ CX43, composed of intercellular channels that enable direct cell‐to‐cell communication, plays a crucial role in stem cell adhesion and migration.^65^ Furthermore, ICAM, also referred to as CD54, mediates interaction with proinflammatory macrophages and boosts the immunosuppressive function of stem cells.^66^ In addition, prior studies demonstrated that ICAM‐deficient mice had delayed skin wound healing.^67^ Intriguingly, in contrast to the inhibited cell proliferation observed in stem cell aggregates, our pressuroid exhibited significantly greater cell proliferation capacity than aggregates generated using conventional techniques. Due to the upregulation of PIEZO1/2 in the pressuroid, *CCND1* was also upregulated, confirming an increase in PCNA (Figure 2).^68^ According to previous research, cell aggregate compaction is crucial for enhancing both cell viability and therapeutic effects. Continuous acoustic pressure generated in the FS device caused single hADSCs in cell suspension to aggregate and became trapped in subaqueous acoustic nodes beginning with the inoculation phase. In contrast to cell aggregation following unidirectional gravity alone (HD group) or temporal pressure with added gravity (FFM group), our FS device promoted ultrafast 3D cell aggregation with intensified cell‐to‐cell contact due to forced omnidirectional intercellular contacts (Figure 2). Unlike aggregates in the HD and FFM groups, rapid cell aggregation in the FS device also induced an identical phase microenvironment around the overall surface of the pressuroid. Since almost half of a cell aggregate in the HD group and FFM group is facing a medium‐air interface or solid surface of cell culture plate closer than the pressuroid,^57^, ^58^ the cellular microenvironment that can transfer supplements, nutrition, and wastes are not identical to subaqueous free‐standing pressuroids.

Furthermore, hADSC aggregates are formed in a free‐standing condition‐induced mechanosensitive gene and protein expression, which can upregulate cell proliferation and angiogenic paracrine factor secretion compared to the conventional method. As depicted in Figures 2 and 4, the expression of the representative mechanosensitive gene and protein PIEZO1/2 was upregulated in FS device‐cultured hADSC aggregates containing cells with extracellular force.^69^, ^70^ Notably, PIEZO1/2 expression is associated with cell proliferation, angiogenic factor paracrine secretion, and cell cycle regulation.^71^, ^72^, ^73^ Indeed, by increasing PIEZO1/2 expression with FS device culture, hADSC aggregates exhibited significantly enhanced *PI3K*, *VEGF*, *CCND1*, and *PCNA* expression. In contrast, *CASPASE‐3* expression decreased (Figure 2). PI3K is involved in cellular processes, including growth, proliferation, differentiation, motility, and survival.^74^ Specifically, the PI3K‐Akt signaling axis controls multiple essential processes in angiogenesis, including endothelial cell survival, migration, formation of capillary‐like structures, and VEGF formation.^27^ Previously, HD and FFM promoted stem cell therapeutic effects by increasing VEGF expression.^75^ Hypoxia‐inducible factor 1 alpha (HIF‐1α) upregulation activated by size‐dependent hypoxic core formation in the aggregate has been reported to be the cause of increased VEGF expression in HD or FFM cultured aggregates.^76^ HIF‐1α induces VEGF upregulation to maintain cell viability under low oxygen and nutrient depletion conditions, which can result in cell death. Therefore, when the size of the aggregates becomes excessively large, the aggregated cells die rapidly. In contrast to HD or FFM culture, the FS device significantly increased *VEGF* gene expression in the absence of HIF‐1α expression, based on PIEZO1/2 and PI3K signaling (Figure 2d). The morphological difference between long‐term HD‐ or FFM cultured cell aggregates (sphere shape) and pressuroid (discoid shape) may have allowed the cells in the core of the pressuroid to transfer oxygen and nutrients via diffusion since the pressuroid is shorter than the cell aggregates previously reported.^77^ In fact, after cell aggregation, the HD 24 h group demonstrated an increase in necrotic cell population due to restricted oxygen and nutrient delivery due to a larger omnidirectional diameter. Since the acoustic pressure at a height greater than 200 μm increased abruptly to 500 Pa, the thickness of hADSCs pressuroids was maintained. This result suggests that pressuroid can be formed easily and without difficulty to prevent excessive acoustic pressure from the FS device (Figure 3a–c). Therefore, the average height of the pressuroid was 200 m, and the average pressure was 12 Pa. (Figure 3a–c). CCND1, which encodes cyclin D1, plays an essential role in the progression of the cell cycle,^78^ and PCNA, known as cyclin D1‐associated protein associated with cell proliferation activity,^79^ were elevated in pressuroid compared to cell aggregates in the HD and FFM group. These findings may be attributable to the rapid cell compaction that promotes harmonious cell‐to‐cell communication. Previously, genetic modification, cellular membrane modification, and scaffold surface modification were used to improve cell‐to‐cell contact for regulating cell functions. However, physical stimulation of 3D cell culture devices has been studied infrequently to elicit cell‐to‐cell contact enhancement.

Pressuroids demonstrated further improvement in cell adhesion and inflammation regulation based on cell–cell communication. FS device induced rapid cell aggregation with elevated adhesion and gap junction proteins, including E‐cadherin, CX43, and ICAM, compared to HD cultured hADSC aggregates (Figure 4b,d). E‐cadherin, one of the most important cell adhesion molecules, increases in stem cell spheroids.^80^ CX43, a ubiquitous gap junction protein, is essential for cell–cell communication (^81^). ICAM is essential for stabilizing cell–cell interaction^82^ and regulating immune responses.^83^

Enhanced CCND1 expression in pressuroids increases cell adhesion as well.^84^ Consequently, the reattachment of hADSCs detached from pressuroids was enhanced (Figure 4I,J). Altogether, these results indicate that enhanced cell–cell interaction in pressuroids affected cell adhesion capacity even after dissociation, which may be related to cell dissociation following in vivo injection of cell aggregates. The pressuroids generated by the FS device facilitated the secretion of cytokines that can recruit and activate immune cells and are related to inflammation. Simultaneously, the secretion of anti‐inflammatory cytokines that reduce inflammation and promote regeneration was higher in pressuroids than in HD cell aggregates (Figure 5). Immune modulation by hADSCs pressuroids is anticipated to maximize in vivo therapeutic efficacy because stem cells injected into a lesion can stimulate host immune cells.^85^ Anti‐inflammatory cytokines stimulate the polarization of macrophages into the M2 type, which inhibits an excessive inflammatory response and promotes tissue regeneration.^86^, ^87^ Compared to other groups, skin wounds in the pressuroid group healed rapidly with reduced fibrosis, increased vascular marker expression, and enhanced skin regeneration‐related ECM marker expressions (Figure 6). Specifically, the angiogenic capacity of transplanted pressuroids stimulated the formation of new blood vessels at wound sites.^88^ Additionally, the ability of pressuroids to modulate the immune system may have been associated with functional skin regeneration rather than fibrosis via enhanced wound epithelization.^89^ The cellular behavior of endothelial cells, fibroblasts, keratinocytes, and macrophages in the host is primarily responsible for skin wound healing.^90^, ^91^, ^92^, ^93^

The intrinsic host cells could have been stimulated to speed up the therapeutic processes by significantly increasing paracrine factor secretions from the pressuroid in comparison to the conventional cell aggregate. Since the angiogenic paracrine factors observed in the pressuroids can cause angiogenesis and cell migration, the participation of microvascular endothelial cells in new blood vessel formation,^93^ fibroblasts to granulation tissue induction,^91^ and keratinocytes to re‐epithelization could have been upregulated compared to other groups (^90^). Moreover, inflammatory‐related paracrine factors secreted by the pressuroids enhanced macrophage recruitment.^94^ Under the influence of an anti‐inflammatory cytokine, the phenotype of flocked macrophages displaying enhanced polarization to anti‐inflammatory M2 macrophages^93^ was enhanced. Subsequently, M2 macrophages may have promoted the migration and proliferation of endothelial cells, fibroblasts, and keratinocytes, thereby enhancing wound healing.^94^ In summary, the transplanted pressuroids upregulated angiogenesis and epithelization in the wounds, thereby enhancing the skin wound healing effect.

## 4 CONCLUSION

This study presents a subaqueous free‐standing cell culture device (FS device) for engineering pressuroid, a stem cell spheroid with a reinforced cytoskeleton. Acoustic pressure was applied via our FS device to efficiently elevate hADSCs and form pressuroid. The FS device induced ultrafast cell compaction elicited mechano‐inductive cellular immune responses and enhanced therapeutic angiogenesis in 3D hADSCs aggregates (pressuroids), distinguishing them from conventional 3D stem cell aggregates. Interestingly, the acoustic pressure generated from the FS device affected the hADSCs in the pressuroid and induced the expression of mechanosensitive genes and proteins. Compared to conventional 3D cell aggregates, enhanced proliferation, angiogenic paracrine factor secretion, cell‐to‐cell adhesion, and immunogenic regulation capacity were observed. Furthermore, the transplantation of pressuroids to skin lesions significantly accelerated and enhanced the therapeutic efficacy of acute wound closure and re‐epithelialization with angiogenesis. Thus, our FS device and pressuroid may suggest a new platform for future biomedical applications involving 3D cell aggregate‐related cell culture systems and cell therapy.

## 5 MATERIALS AND METHODS

### 5.1 Cell culture device

The piezoelectric actuator used in this study was made of lead zirconate titanate (PZT) and fabricated as a 20 mm in diameter, 1.3 mm thick, round disc with 1800 pF capacitance and 1.6 MHz resonance frequency. The details of the actuator are shown in Figure S4. The piezoelectric actuator was installed at the bottom of the cell culture vessel, and the reflector was positioned about the upper side. As the sidewall of the cell culture vessel, a borosilicate tube with an outer diameter of 50 mm and a wall thickness of 5 mm was used. Both the top and bottom sides of the vessel tube were plugged in with O‐ring sealed actuator and reflector components. In order to control the distance between the actuator and reflector to tune the resonance condition of acoustic waves, the holder of the reflector was designed to be movable. In order to maintain a temperature of approximately 36°C in the cell culture vessel, cooling water was circulated through the aluminum frame containing heat sources such as the actuator and the driving circuit.

### 5.2 Acoustic pressure and particle tracing calculations

The Acoustics and Particle tracing module of COMSOL Multiphysics® 5.5 was used to calculate the spatial distribution of an acoustic standing wave generated by a piezoelectric actuator. Subsequently, the trajectories of small particles in the cell culture vessel containing an acoustic standing wave and the trajectories of a large particle in the acoustic standing wave formed in the cell culture vessel. In all calculations, PZT‐5A from the COMSOL library was considered an actuator material, and water was specified as the medium filling in the cell culture device. In addition, we assumed that all particles have a spherical shape and are composed of rigid matter, and we set all measurement parameters according to the actual cell culture apparatus. Figure S5 and Data [Link], [Link] summarize the modeling details for the above three calculations.

### 5.3 Cell culture

hADSCs were purchased from Lonza (Walkersville, MD, USA) and cultured in cell culture dishes (Corning, Steuben, NY, USA) containing Dulbecco's modified Eagle's medium (Gibco BRL, Gaithersburg, MD, USA), supplemented with 10% (v/v) fetal bovine serum (FBS; Gibco BRL) and 1% (v/v) penicillin–streptomycin (PS; Gibco BRL), in a 5% CO_2_ cell incubator at 37°C. The culture medium was changed every second day, and the hADSCs in passages 4–7 were used in subsequent experiments.

### 5.4 Real‐time polymerase chain reaction

TRIzol (Ambion, Austin, TX, USA), chloroform (Sigma‐Aldrich), and 75% (v/v) ethanol (Sigma‐Aldrich, in water) were used according to the manufacturer's instructions to isolate total RNA from cells. Complementary DNA was synthesized via reverse transcription using 1.5‐μg pure total RNA and the Primescript RT Master Mix (TaKaRa, Kusatsu, Japan). Subsequently, qRT‐PCR was performed using the SsoAdvanced Universal SYBR Green Supermix (Bio‐Rad, Hercules, CA, USA) and CFX Connect™ Real‐time PCR Detection System (Bio‐Rad). Analyzing the relative expression level using the 2^−∆∆Ct^ method. Furthermore, glyceraldehyde 3‐phosphate dehydrogenase (GAPDH, in vitro) and beta‐actin (β‐actin, in vivo) served as the internal controls.

### 5.5 Immunocytochemistry

The 3D hADSCs were fixed at room temperature with 4% paraformaldehyde (Biosesang, Sungnam, Korea) for 10 min. Using a microscope (CKX53; Olympus, Tokyo, Japan), the morphology of the pressuroid was analyzed (FS 6 h). The fixed 3D hADSCs were embedded in a compound with optimal cutting temperature (O.C.T. compound, Scigen Scientific, Gardena, CA, USA). After freezing, samples were sectioned at −20°C into 10 μm sections. These sections containing spheroids were stained immunocytochemically to visualize E‐cadherin and PIEZO2 with anti‐E‐cadherin (Abcam), anti‐PIEZO2 (Abcam), and fluorescein (FITC)‐conjugated secondary antibodies (Jackson ImmunoResearch Laboratories, West Grove, PA, USA). In addition, these sections were stained with TRITC‐phalloidin, which contained a mounting medium (VECTASHIELD H‐1600; Vector, Burlingame, CA, USA) for F‐actin staining. Subsequently, they were counterstained with 4′,6‐diamidino‐2‐phenylindole (DAPI, Vector) and examined under a fluorescence microscope (DFC 3000G; Leica, Wetzlar, Germany).

### 5.6 Western blot analysis

In order to prepare protein samples, cells or tissues were extracted in radioimmunoprecipitation assay buffer (Sigma‐Aldrich). The bicinchoninic acid assay was conducted to determine protein concentration (Thermo Scientific). Proteins were boiled for 5 min at 100°C in 4× Laemmli sample buffer (Thermo Scientific) containing β‐mercaptoethanol, and amounts of proteins were loaded onto a 10% SDS‐PAGE gel. Next, the separated proteins were transferred to polyvinylidene fluoride membranes and blocked for 1 h at room temperature with 1× TBS‐T, containing 5% skim milk was the next step. The membranes were incubated overnight with primary antibodies, washed with 1× TBS‐T, and incubated for 1 h at room temperature with secondary antibodies. After TBS‐T washes, protein bands were visualized with ECL reagent WESTSAVE UP (ABfrontier, Seoul, Korea), and membranes were exposed to x‐ray films. ImageJ software (National Institutes of Health, Bethesda, MD, USA) was used to analyze band expression. GAPDH was implemented as an internal control.

### 5.7 Apoptosis assays

Following the manufacturer's instructions, an apoptosis assay was performed using a FITC annexin V apoptosis detection kit with 7‐AAD (BD Biosciences, San Diego, CA, USA). hADSCs in 3D were dissociated with trypsin–EDTA (Gibco BRL). The cells were incubated for 15 min at room temperature and in the dark with FITC Annexin V and 7‐AAD. Following staining, the cells were added to an annexin V binding buffer and analyzed using a flow cytometer (MACSQuant® VYB, Miltenyi Biotec, Bergisch Gladbach, Germany). Annexin V and 7‐AAD bind phosphatidylserine (PS) and cell nuclei, respectively, of nonviable cells. Annexin V−/7AAD− cells were thought to be alive, annexin V+/7AAD− cells were considered early apoptotic cells, and annexin V+/7AAD+ cells were considered late apoptotic or early necrosis cells.

### 5.8 Re‐adhesion analysis

Cell aggregates were dissociated with trypsin–EDTA (Gibco BRL) for the re‐adhesion test and reseeded in cell culture plates. Following an incubation period of 1 or 3 h, the plates were washed with phosphate buffer saline (PBS) to remove unattached cells. Subsequently, using the Cell Counting Kit‐8, the relative cell adhesion rate of the cells following treatment was determined (CCK‐8; Dojindo Molecular Technologies, Inc., Kumamoto, Japan). The control group consisted of cells that were detached from HD 24 h and reattached for 1 h. Two hours after incubation with the CCK‐8 solution at 37°C, the optical density of each well was measured using a microplate reader (450‐nm; Tecan, Mannedorf, Switzerland).

### 5.9 Human cytokine array

Proteome profiler human cytokine array kit (R&D Systems, Minneapolis, MN, USA) was used to analyze the expression of immunomodulatory cytokines in pressuroids according to the manufacturer's protocol. ImageJ software (National Institutes of Health, Bethesda, MD, USA) was used to quantify pixel density in each spot, and the average signal was calculated for the duplicate spots.

### 5.10 THP‐1 cell culture and differentiation

American Type Culture Collection (Manassas, VA, USA) THP‐1 cells were purchased and cultured in cell culture flasks (Corning) with RPMI‐1640 medium (11875, Gibco BRL), supplemented with 10% (v/v) FBS (Gibco BRL) and 1% (v/v) PS (Gibco BRL), in a 5% CO_2_ cell incubator at 37°C. Every second day, the nutrient medium was replaced. THP‐1 cells at passages 2–5 were used in the experiments. THP‐1 cells were differentiated with 100 nM ml of phorbol 12‐myristate 13‐acetate (PMA, Sigma‐Aldrich) for 2 days. Cells were washed with PBS and polarized toward the M1 or M2 phenotype by incubation for 2 days with a sample medium. Next, using a microscope, THP‐1 cells were observed after incubation (DFC 3000G).

### 5.11 Wound healing and repair

Xylazine (10 mg/kg) and ketamine (100 mg/kg) were injected intraperitoneally into athymic mice (6 weeks old; body weight, 20 g; Orient Bio Inc., Sungnam, Korea) to induce anesthesia. The mid‐dorsal area of the skin of each mouse was sliced to make a full‐thickness skin wound (2.0 × 2.0 cm^2^). Next, the Institutional Animal Care and Use Committee of SKKU authorized all animal treatments and experimental procedures (SKKUIACUC2020‐06‐11‐1). Mice with skin wounds were randomly divided into three experimental groups (*n* = 6 per group): NT; HD‐treated group (HD; 0.75 × 10^6^ cells suspended in 200‐μl per mouse); and pressuroid‐treated group (pressuroid; 0.75 × 10^6^ cells suspended in 200‐μl per mouse). The NT group undergoing only wound modeling served as the negative control (NT). Tegaderm™ (3M Health Care, St. Paul, MN, USA), which is a common dressing material, was applied to all groups.^49^ After initial treatments, the wound healing process was observed for up to 14 days. The percentage of the initial wound area was used to calculate wound healing: $\frac{\text{wound area}\mathrm{at}a\text{time}}{\text{initial wound area}}\times 100\%$.

All samples were gathered in order to accurately compare wound healing between the various groups. In order to compare the wound healing process in each group, whole tissues from the dorsal wound area were also extracted for analysis. At 14 days, tissues in the wound region were collected, and then homogenized with homogenizer. TRIzol (Ambion), chloroform (Sigma‐Aldrich), and 75% (v/v) ethanol (Sigma‐Aldrich, in water) were used to isolate total RNA from tissues according to the manufacturer's instructions.

### 5.12 Histology and immunohistochemistry

Several histological analyses were performed on the wound tissues of athymic mice. Skin tissue samples were embedded in O.C.T. compound (Tissue‐Plus®; Scigen), frozen, and cut into 10 μm sections at −23°C. Under a light microscope, hematoxylin and eosin (H&E) and Masson's trichrome (MT) stains were used to evaluate overall tissue regeneration (CKX53; Olympus). To visualize protein expression and wound repair, sections were immunostained with anti‐SM‐α antibody (Abcam), anti‐CD31 antibody (Abcam), antilaminine antibody (Abcam), antiinvolucrin antibody (BioLegend, San Diego, CA, USA), and a fluorescein isothiocyanate‐conjugated secondary antibody (Jackson ImmunoResearch Laboratories). The sections were counterstained with DAPI (VECTASHIELD H‐1500, Vector) and examined under a fluorescence microscope (Leica).

### 5.13 Statistical analysis

All statistical analyses utilized GraphPad Prism 7 software. In all experiments, triplicate data were analyzed using a one‐way analysis of variance with the Bonferroni test. Additionally, two independent samples were compared using a two‐tailed Student's *t*‐test. The statistical significance threshold was set at *p* < 0.05. For all quantitative analyses, results were presented as the mean ± standard deviation.

## AUTHOR CONTRIBUTIONS

**Gwang‐Bum Im:** Conceptualization (equal); investigation (equal); methodology (equal); visualization (equal); writing – original draft (equal); writing – review and editing (equal). **Yu‐Jin Kim:** Conceptualization (equal); investigation (equal); methodology (equal); visualization (equal); writing – original draft (equal); writing – review and editing (equal). **Suk Ho Bhang:** Conceptualization (equal); methodology (equal); supervision (equal); writing – original draft (equal); writing – review and editing (equal). **Tae Il Lee:** Conceptualization (equal); methodology (equal); supervision (equal); writing – review and editing (equal).

## CONFLICT OF INTEREST

The authors declare no conflict of interest.

### PEER REVIEW

The peer review history for this article is available at https://publons.com/publon/10.1002/btm2.10438.

## Supporting information


**Fig. S1.** (A) Relative *Piezo1* expression compared to single‐cell group (*n* = 6, *p < 0.05, and **p < 0.001 compared with the single‐cell group, #*p* < 0.05 and ##*p* < 0.001 compared to each other, N.S.: not statistically different with single‐cell group). (B) Summary of statistically difference in Figure 2B–G between groups (*p < 0.05 and **p < 0.001 compared to each other, −: not statistically different with each other)
**Figure S2**. (A) Relative mRNA expression of *VEGF*, *COX‐2*, and *IL‐10* in HD 6 h and FS 6 h (*n* = 3, *p < 0.05 compared with HD 6 h group). (B) Immunostaining for CX43^+^ (yellow), F‐actin (red), and DAPI (blue) in HD 6 h and FS 6 h. Scale bars indicate 250 μm. (C) Representative optical images of 3D cell aggregation after pipetting 10 times. Scale bars indicate 250 μm.
**Figure S3**. Expression of immunomodulation‐related genes (*IL‐1β*, *IL‐6*, and *CXCL12*) in pressuroid group analyzed using qRT‐PCR. The HD 24 h group was served as the control group (*p < 0.05, compared to HD 24 h group, *n* = 5).
**Figure S4**. The material and physical properties of piezoelectric actuator used in this work. The actuator was designed to operate in d_33_ mode as the electrode configuration shown in the camera images.
**Figure S5**. The modeling parameters for calculating the spatial distribution of acoustic pressure and trajectories of particles in the cell culture vessel. For modeling, the Acoustic Module and the Particle Tracing Module with COMSOL Multiphysics® 5.5 were used.
**Table S1**. qRT‐PCR primer sequences. Primer sequences were verified through BLAST.

**Movie S1.** Total acoustic pressure field (Pa) Particle trajectories.

**Data S1.** Supporting document for Acoustic pressure Calculation

**Data S2.** Supporting document for single‐cell calculation

**Data S3.** Supporting document for spheroid calculation

## Data Availability

All data are available in the main text or the supplementary materials.
