# Supplementary material for: Subaqueous free‐standing 3D cell culture system for ultrafast cell compaction, mechano‐inductive immune control, and improving therapeutic angiogenesis
Source: Bioeng Transl Med. 2022 Oct 28;8(2):e10438. doi: 10.1002/btm2.10438 (PMC10013761; doi:10.1002/btm2.10438)
Supplement: Supplementary file 1 — Fig. S1. (A) Relative Piezo1 expression compared to single‐cell group (n = 6, *p < 0.05, and **p < 0.001 compared with the single‐cell group, #p < 0.05 and ##p < 0.001 compared to each other, N.S.: not statistically different with single‐cell group). (B) Summary of statistically difference in Figure 2B–G between groups (*p < 0.05 and **p < 0.001 compared to each other, −: not statistically different with each other) Figure S2. (A) Relative mRNA expression of VEGF, COX‐2, and IL‐10 in HD 6 h and FS 6 h (n = 3, *p < 0.05 compared with HD 6 h group). (B) Immunostaining for CX43+ (yellow), F‐actin (red), and DAPI (blue) in HD 6 h and FS 6 h. Scale bars indicate 250 μm. (C) Representative optical images of 3D cell aggregation after pipetting 10 times. Scale bars indicate 250 μm. Figure S3. Expression of immunomodulation‐related genes (IL‐1β, IL‐6, and CXCL12) in pressuroid group analyzed using qRT‐PCR. The HD 24 h group was served as the control group (*p < 0.05, compared to HD 24 h group, n = 5). Figure S4. The material and physical properties of piezoelectric actuator used in this work. The actuator was designed to operate in d33 mode as the electrode configuration shown in the camera images. Figure S5. The modeling parameters for calculating the spatial distribution of acoustic pressure and trajectories of particles in the cell culture vessel. For modeling, the Acoustic Module and the Particle Tracing Module with COMSOL Multiphysics® 5.5 were used. Table S1. qRT‐PCR primer sequences. Primer sequences were verified through BLAST. [file BTM2-8-e10438-s002.docx]

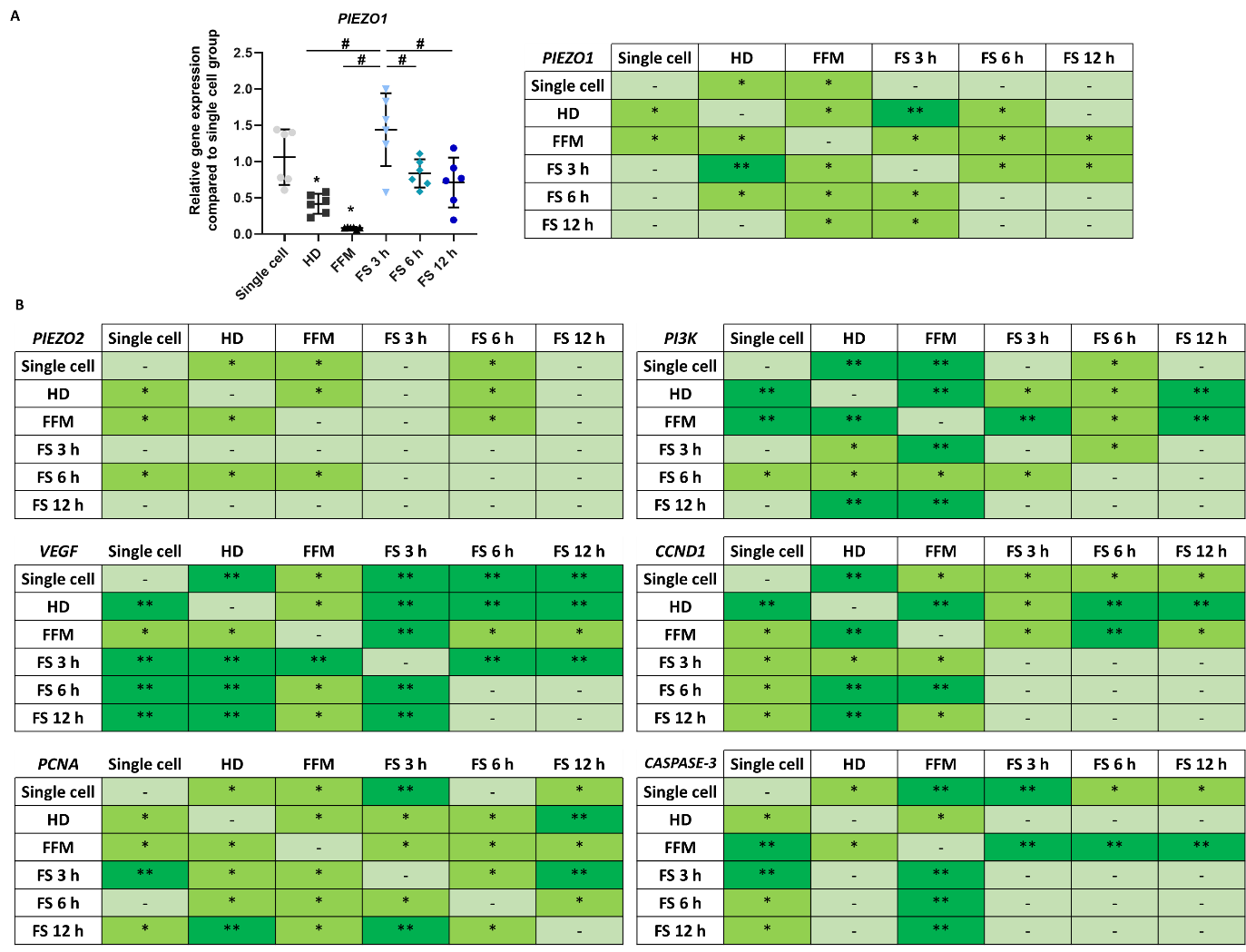


Fig. S1.

(A) Relative *Piezo1* expression compared to single-cell group (n = 6, **P* < 0.05, and ***P* < 0.001 compared with the single-cell group, #*P* < 0.05 and ##*P* < 0.001 compared to each other, N.S.: not statistically different with single-cell group). (B) Summary of statistically difference in figure 2 B–G between groups (**P* < 0.05 and ***P* < 0.001 compared to each other, -: not statistically different with each other)


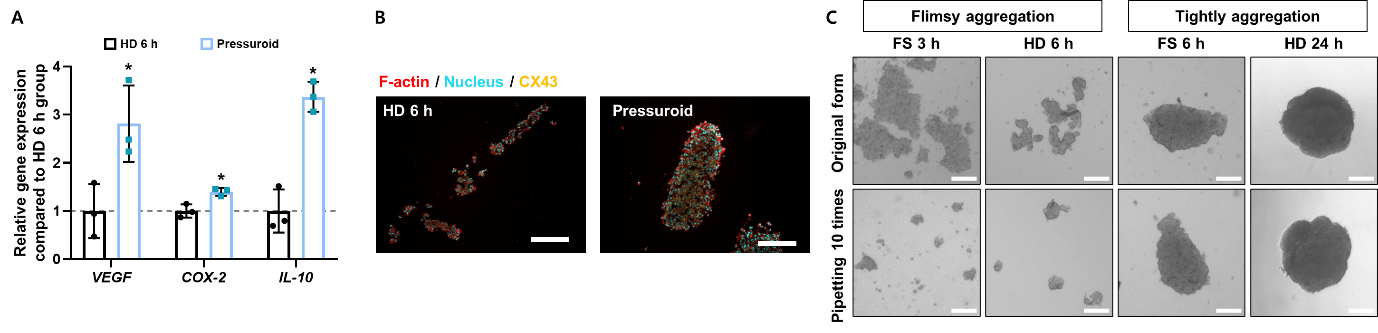


Fig. S2.

(A) Relative mRNA expression of *VEGF*, *COX-2*, and *IL-10* in HD 6 h and FS 6 h (n = 3, **P* < 0.05 compared with HD 6 h group). (B) Immunostaining for CX43^+^ (yellow), F-actin (red), and DAPI (blue) in HD 6 h and FS 6 h. Scale bars indicate 250 μm. (C) Representative optical images of 3D cell aggregation after pipetting ten times. Scale bars indicate 250 μm.


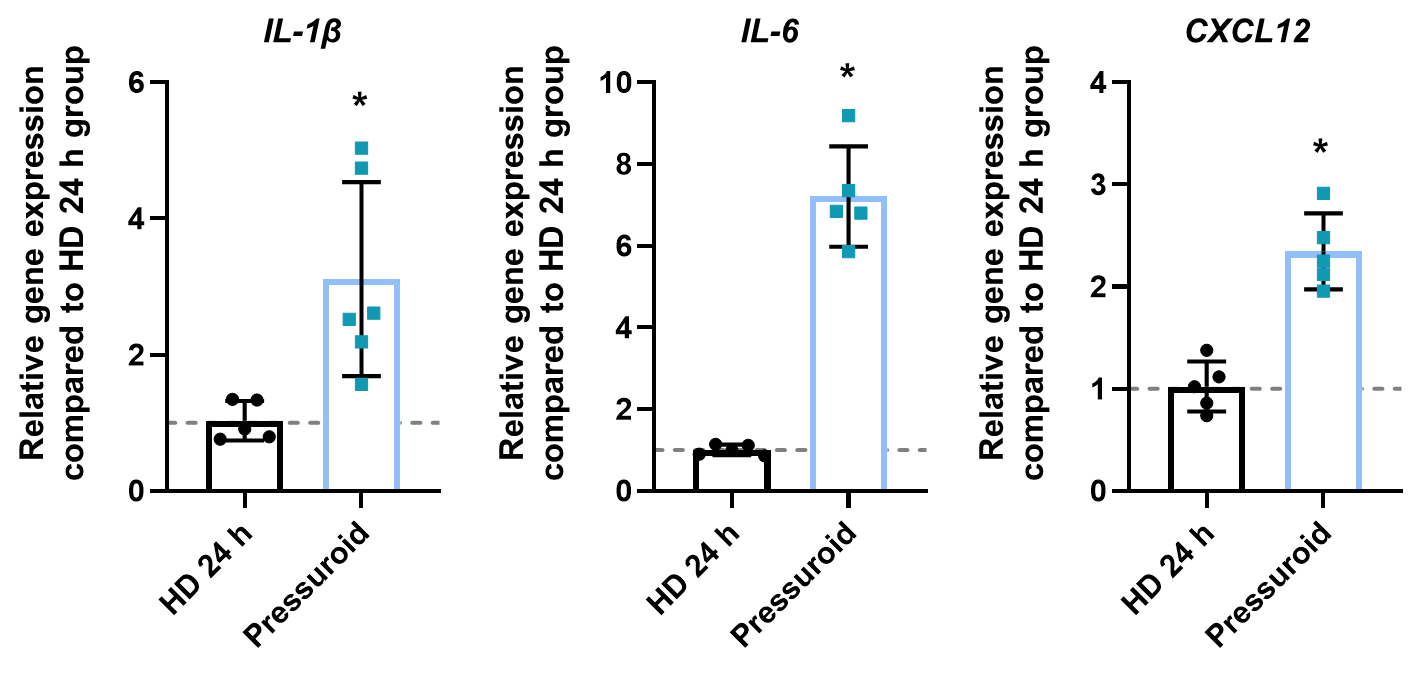


Fig. S3.

Expression of immunomodulation-related genes (*IL-1β*, *IL-6*, and *CXCL12*) in pressuroid group analyzed using qRT-PCR. The HD 24 h group was served as the control group (*P < 0.05, compared to HD 24 h group, n = 5).


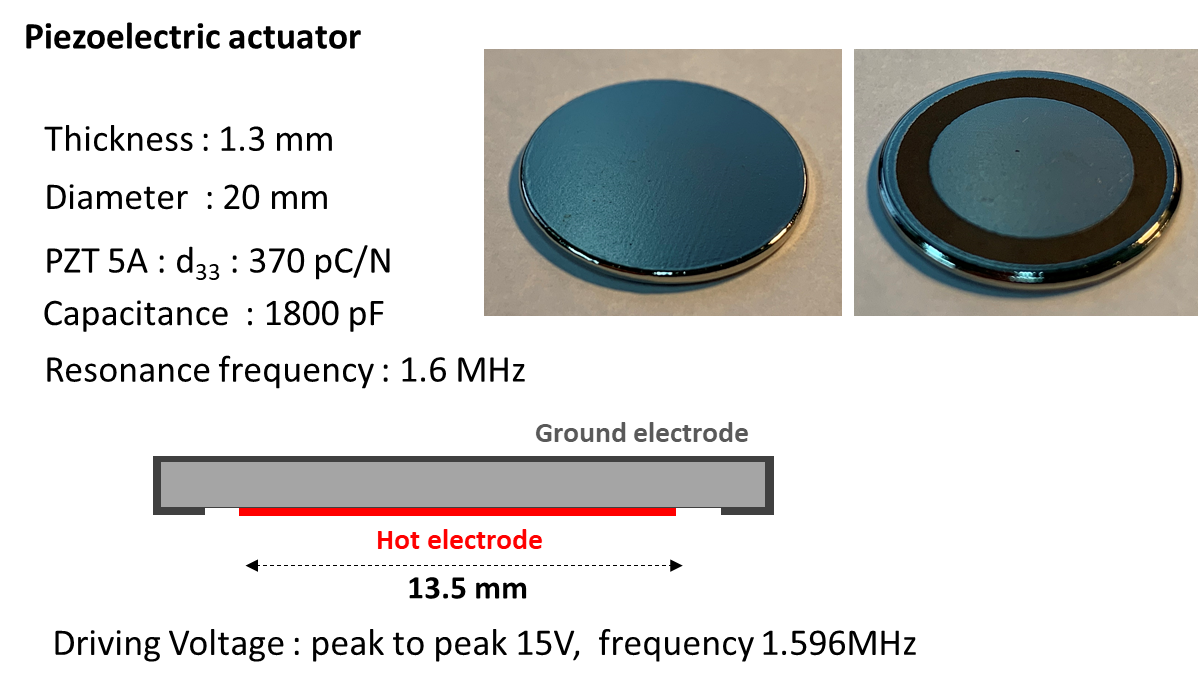


Fig. S4.

The material and physical properties of piezoelectric actuator used in this work. The actuator was designed to operate in d_33_ mode as the electrode configuration shown in the camera images.


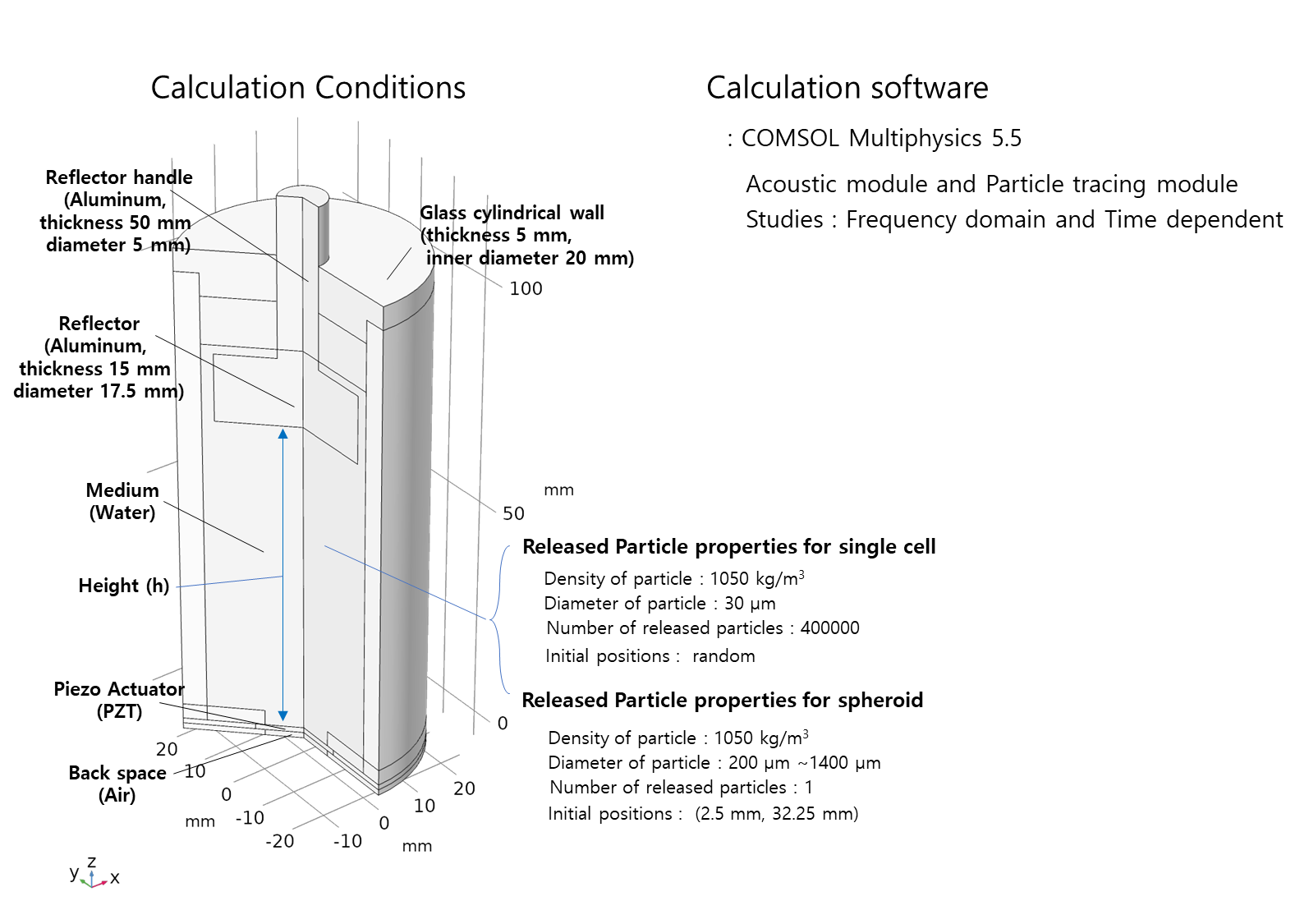


Fig. S5.

The modeling parameters for calculating the spatial distribution of acoustic pressure and trajectories of particles in the cell culture vessel. For modeling, the Acoustic Module and the Particle Tracing Module with COMSOL Multiphysics® 5.5 were used.

Table S1.

qRT-PCR primer sequences. Primer sequences were verified through BLAST.

| Gene | Primer | Sequence (5′-3′) |
| --- | --- | --- |
| *Human GAPDH* | Forward | GTC GGA GTC AAC GGA TTT GG |
|  | Reverse | GGG TGG AAT CAA TTG GAA CAT |
| *Human PIEZO2* | Forward | CGG CCT AGG CTC CGT AAA AT |
|  | Reverse | GGG CTC GCT TGA TGC TAA AC |
| *Human PI3K* | Forward | CAA AGC CGA GAA CCT ATT GCG AG |
|  | Reverse | GTT TGA CTT CGC CAT CTA CCA C |
| *Human VEGF* | Forward | GAG GGC AGA ATC ATC ACG AAG T |
|  | Reverse | CAC CAG GGT CTC GAT TGG AT |
| *Human CCND1* | Forward | GAG ACC ATT CCC CTG ACT GC |
|  | Reverse | CCA TTT GCA GCA ACT CCT CG |
| *Human PCNA* | Forward | CCT GCT GGG ATA TTA GCT CCA |
|  | Reverse | CAG CGG TAG GTG TCG AAG C |
| *Human CASPASE-3* | Forward | CCT GGT TAT TAT TCT TGG CGA AA |
|  | Reverse | GCA CAA AGC GAC TGG ATG AA |
| *Human E-CADHERIN* | Forward | CCC ACC ACG TAC AAG GGT C |
|  | Reverse | CTG GGG TAT TGG GGG CAT C |
| *Human CX43* | Forward | TCT CTC ATG TGC GCT TCT GG |
|  | Reverse | TGA CAC CAT CAG TTT GGG CA |
| *Human ICAM* | Forward | TCT TCC TCG GCC TTC CCA TA |
|  | Reverse | AGG TAC CAT GGC CCC AAA TG |
| *Human HGF* | Forward | GAT GGC CAG CCG AGG C |
|  | Reverse | TCA GCC CAT GTT TTA ATT GCA |
| *Human IL-1b* | Forward | AAT TTG AGT CTG CCC AGT TCC C |
|  | Reverse | AGT CAG TTA TAT CCT GGC CGC C |
| *Human IL-6* | Forward | GCA CTG GCA GAA AAC AAC CT |
|  | Reverse | TCA AAC TCC AAA AGA CCA GTG A |
| *Human CXCL12* | Forward | TGC CAG AGC CAA CGT CAA G |
|  | Reverse | CAG CCG GGC TAC AAT CTG AA |
| *Human TNF-α* | Forward | TCT TCT CGA ACC CCG AGT GA |
|  | Reverse | CCT CTG ATG GCA CCA CCA G |
| *Human MRC-1* | Forward | CCA AAC GCC TTC ATT TGC CA |
|  | Reverse | ACC TTC CTT GCA CCC TGA TG |
| *Human CD80* | Forward | TCA GAA GTG GAG TCT TAC CCT G |
|  | Reverse | CCT GGG TCT CCA AAG GTT GT |
| *Human CD83* | Forward | AGC AGC CAA AAT GGA TCC CC |
|  | Reverse | TGA AGT TAG CAG AGA GCA GGA |
| *Mouse β-actin* | Forward | GGC TGT ATT CCC CTC CAT CG |
|  | Reverse | CCA GTT GGT AAC AAT GCC ATG T |
| *Mouse Sm- α actin* | Forward | CAG GCA TGG ATG GCA TCA ATC AC |
|  | Reverse | ACT CTA GCT GTG AAG TCA GTG TCG |
| *Mouse Cd31* | Forward | CAA ACA GAA ACC CGT GGA GAT G |
|  | Reverse | ACC GTA ATG GCT GTT GGC TTC |
| *Mouse Involucrin* | Forward | CCTGTGAGTTTGTTTGGTCTACA |
|  | Reverse | GAACCACAGCTGGAACAGTC |
| *Mouse Keratin 14* | Forward | CCG ACC TGG AGA TGC AGA TT |
|  | Reverse | GCC ACC TCC TCG TGG TTC |
| *Mouse Keratin 10* | Forward | CAG TTC TCT TCC TCC CGC AG |
|  | Reverse | GAG CTC CCA CGG CTA AAA GA |
| *Mouse Col 4* | Forward | TCA TTA GCA GGT GTG CGG TT |
|  | Reverse | GTT AGG GCA CTG CGG AAT CT |
| *Mouse Col 1* | Forward | GGC AAC AGT CGC TTC ACC TA |
|  | Reverse | AGG CTA AAC CAG ATG CCC AA |
