# Supplementary material for: Subaqueous free‐standing 3D cell culture system for ultrafast cell compaction, mechano‐inductive immune control, and improving therapeutic angiogenesis
Source: Bioeng Transl Med. 2022 Oct 28;8(2):e10438. doi: 10.1002/btm2.10438 (PMC10013761; doi:10.1002/btm2.10438)
Supplement: Supplementary file 4 — Data S2. Supporting document for single‐cell calculation [file BTM2-8-e10438-s004.pdf]

Acoustic 3D Cell Culture System(single cell)

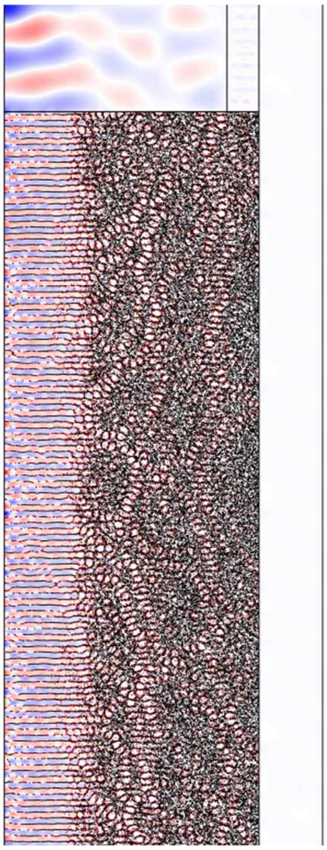

1 Global Definitions

GLOBAL SETTINGS

Name	V2 Acoustic 3D Cell Culture System single cell.mph
Version	COMSOL Multiphysics 5.5 (Build: 359)

USED PRODUCTS

COMSOL Multiphysics
Acoustics Module
Particle Tracing Module

1.1 PARAMETERS

PARAMETERS 1

Name	Expression	Value	Description
f0	1.596[MHz]	1.596E6 Hz	
T	1.2[mm]	0.0012 m	
c0	1530[m/s]	1530 m/s	
H	67.52[mm]	0.06752 m	
V0	15[V]	15 V	
d0	30[um]	3E−5 m	

2 Component 1

2.1 DEFINITIONS

2.1.1 Variable Utilities

Participation factors 1

Tag	mpf1

CENTER OF ROTATION

Description	Value
Center of rotation	Center of mass

2.1.2 Coordinate Systems

Boundary System 1

Coordinate system type	Boundary system
Tag	sys1

COORDINATE NAMES

First	Second	Third
t1	to	n

Base vector system zx

Coordinate system type	Base vector system
Tag	comp1_zx_sys

COORDINATE NAMES

First	Second	Third
x1	x2	x3

BASE VECTORS

	r	z
x1	0	1
x3	1	0

SIMPLIFICATIONS

Description	Value
Assume orthonormal	On

Base vector system xz

Coordinate system type	Base vector system
Tag	comp1_xz_sys

COORDINATE NAMES

First	Second	Third
x1	x2	x3

BASE VECTORS

	r	z
x1	1	0
x3	0	1

SIMPLIFICATIONS

Description	Value
Assume orthonormal	On

2.2 GEOMETRY 1

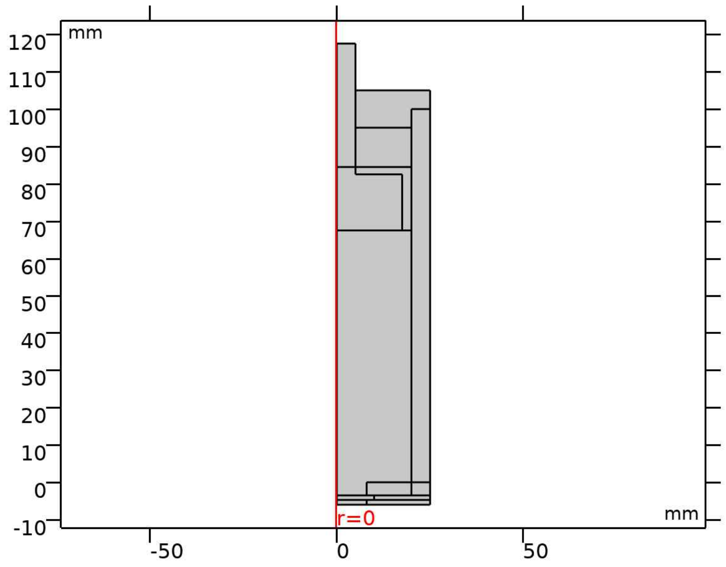

Geometry 1

UNITS

Length unit	mm
Angular unit	deg

2.3 MATERIALS

2.3.1 Lead Zirconate Titanate (PZT-5A)

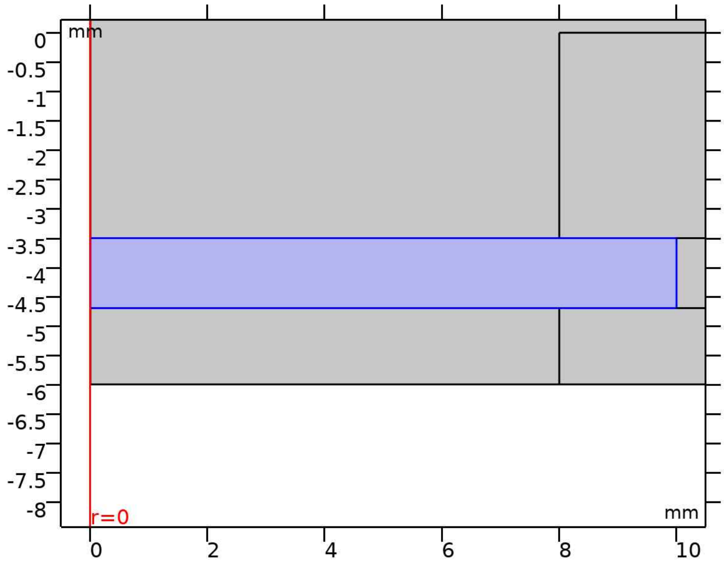

Lead Zirconate Titanate (PZT-5A)

SELECTION

Geometric entity level	Domain
Selection	Geometry geom1: Dimension 2: Domain 2

2.3.2 Glass (quartz)

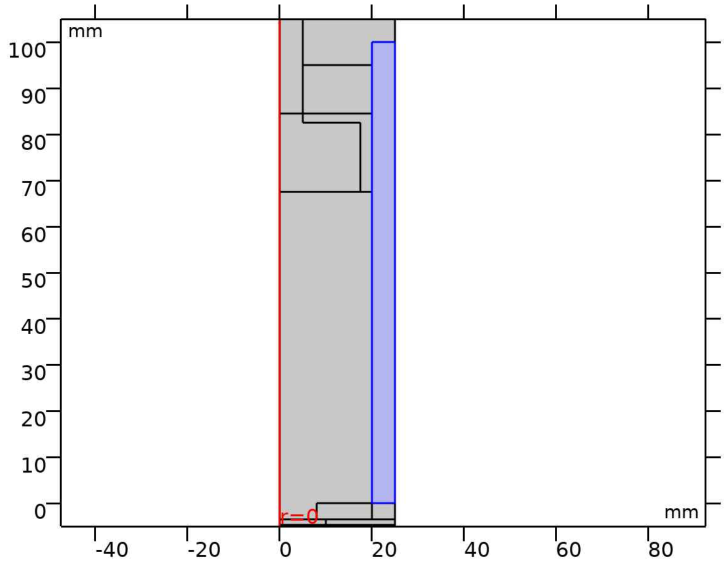

Glass (quartz)

SELECTION

Geometric entity level	Domain
Selection	Geometry geom1: Dimension 2: Domain 13

2.3.3 Aluminum

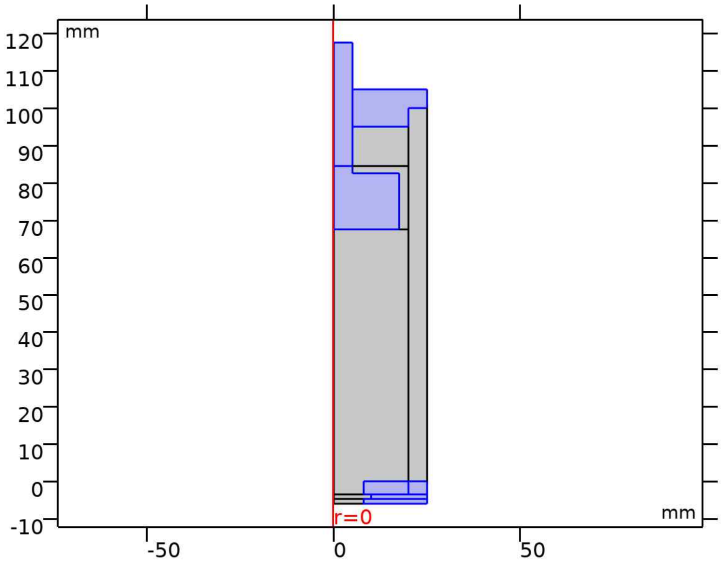

Aluminum

SELECTION

Geometric entity level	Domain
Selection	Geometry geom1: Dimension 2: Domains 4–5, 8–12

2.3.4 Water, liquid

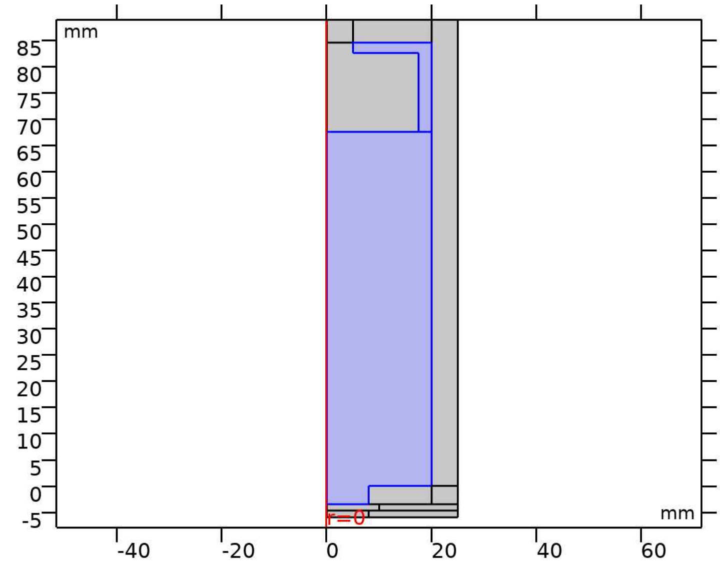

Water, liquid

SELECTION

Geometric entity level	Domain
Selection	Geometry geom1: Dimension 2: Domains 3, 6

2.3.5 Air

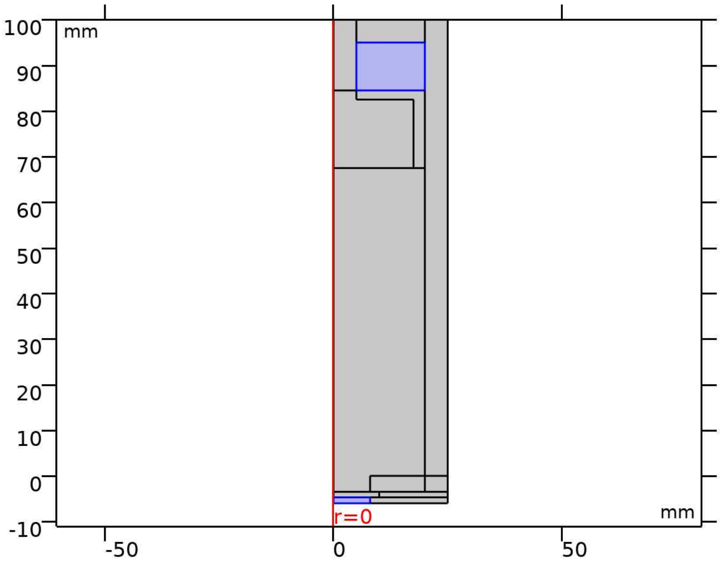

Air

SELECTION

Geometric entity level	Domain
Selection	Geometry geom1: Dimension 2: Domains 1, 7

2.4 SOLID MECHANICS

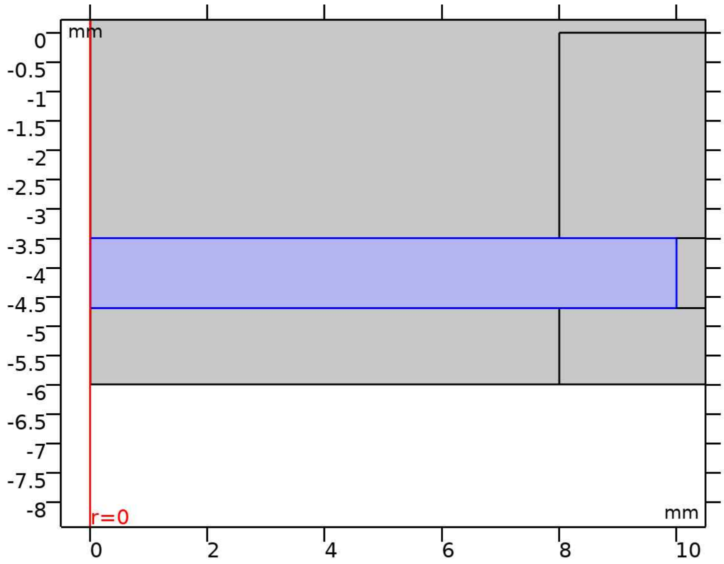

Solid Mechanics

EQUATIONS

$$-\rho\omega^2\mathbf{u} = \nabla\cdot S + \mathbf{F}_V e^{i\phi}, \quad -ik_Z = \lambda$$

FEATURES

Linear elastic material 1
Axial Symmetry 1
Free 1
Initial Value 1
Piezoelectric Material 1

2.4.1 Linear elastic material 1

EQUATIONS

$$-\rho\omega^2\mathbf{u} = \nabla\cdot S + \mathbf{F}_V e^{i\phi}, \quad -ik_Z = \lambda$$

$$S = S_{ad} + \mathbf{C}:\epsilon_{el}, \quad \epsilon_{el} = \epsilon - \epsilon_{inel}$$

$$\epsilon_{inel} = \epsilon_0 + \epsilon_{ext} + \epsilon_{th} + \epsilon_{hs} + \epsilon_{pl} + \epsilon_{cr} + \epsilon_{vp}$$

$$S_{ad} = S_0 + S_{ext} + S_q$$

$$\epsilon = \frac{1}{2}\left[(\nabla\mathbf{u})^T + \nabla\mathbf{u}\right]$$

$$\mathbf{C} = \mathbf{C}(E,\nu)$$

2.4.2 Piezoelectric Material 1

EQUATIONS

$$-\rho\omega^2\mathbf{u} = \nabla\cdot S + \mathbf{F}_V e^{i\phi}, \quad -ik_z = \lambda$$

$$\nabla\cdot\mathbf{D} = \rho_v$$

$$S = S_0 + \mathbf{C} : \epsilon_{el} - \mathbf{E}\cdot \mathbf{e}, \quad \epsilon_{el} = \epsilon - \epsilon_{inel}$$

$$S_{ad} = S_0 + S_{ext} + S_q$$

$$\epsilon_{inel} = \epsilon_0 + \epsilon_{th}$$

$$\mathbf{C} = \mathbf{C}(c_E), \quad \mathbf{e} = \mathbf{e}(e_{ES})$$

$$\epsilon = \frac{1}{2}\left[(\nabla\mathbf{u})^T + \nabla\mathbf{u}\right]$$

$$\mathbf{D} = \mathbf{D}_r + \mathbf{e} : \epsilon_{el} + \epsilon_{0,vac}\epsilon_{rS}\cdot\mathbf{E}$$

2.5 ELECTROSTATICS

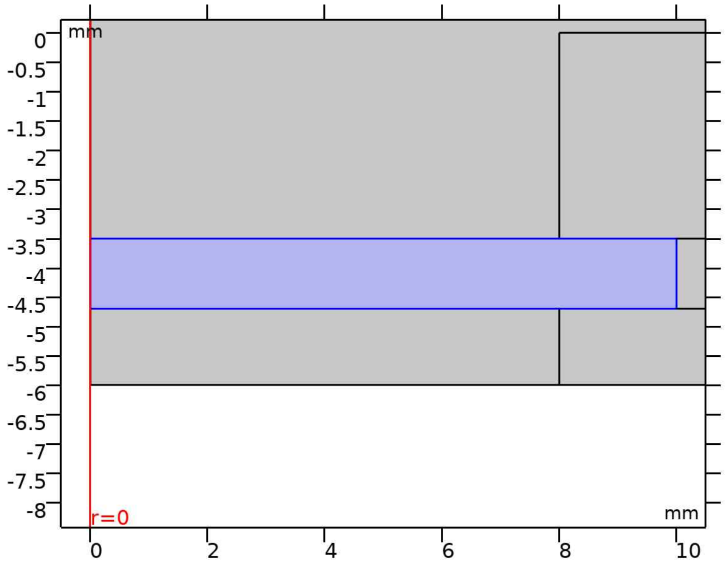

Electrostatics

EQUATIONS

$$\nabla\cdot\mathbf{D} = \rho_v$$

$$\mathbf{E} = -\nabla V$$

FEATURES

Charge Conservation 1
Axial Symmetry 1
Zero Charge 1
Initial Value 1
Charge Conservation, Piezoelectric 1

Electric Potentential 1
Ground 1

2.5.1 Charge Conservation 1

EQUATIONS

$$\mathbf{E} = -\nabla V$$
$$\nabla \cdot (\epsilon_0 \epsilon_r \mathbf{E}) = \rho_v$$

2.5.2 Zero Charge 1

EQUATIONS

$$\mathbf{n} \cdot \mathbf{D} = 0$$

2.5.3 Charge Conservation, Piezoelectric 1

EQUATIONS

$$\mathbf{E} = -\nabla V$$
$$\nabla \cdot \mathbf{D} = \rho_v$$

2.5.4 Electric Potentential 1

EQUATIONS

$$V = V_0$$

2.5.5 Ground 1

EQUATIONS

$$V = 0$$

2.6 PRESSURE ACAUSTICS, FREQUENCY DOMIAN

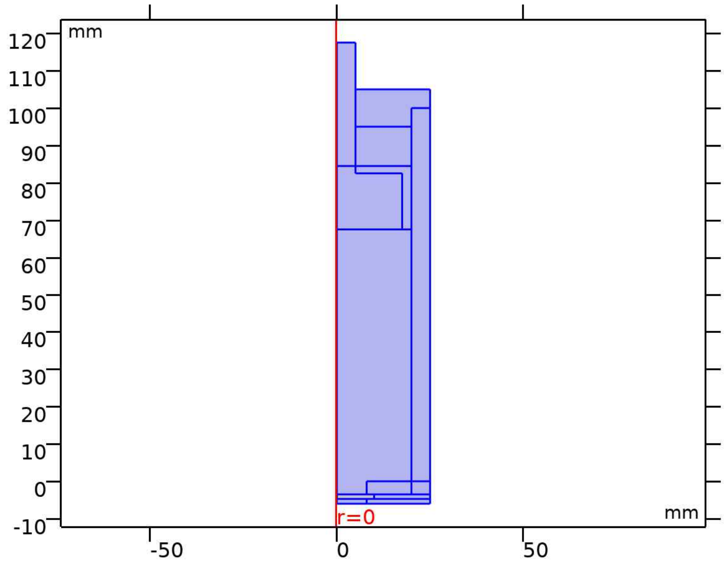

Pressure Acaustics, Frequency Domian

EQUATIONS

$$\nabla \cdot \left(-\frac{1}{\rho_c} (\nabla p_t - \mathbf{q}_d) \right) - \frac{k_{eq}^2 p_t}{\rho_c} = Q_m$$

$$p_t = p + p_b$$

$$k_{eq}^2 = \left(\frac{\omega}{c_c} \right)^2 - k_m^2$$

FEATURES

Pressure Acaustics 1
Axial Symmetry 1
Sound Hard Boundary (Wall) 1
Initial Value 1
Plane Wave Radiation 1
Sound Soft Boundary 1

2.6.1 Pressure Acaustics 1

EQUATIONS

$$\nabla \cdot \left(-\frac{1}{\rho_c} (\nabla p_t - \mathbf{q}_d) \right) - \frac{k_{eq}^2 p_t}{\rho_c} = Q_m$$

$$p_t = p + p_b$$

$$k_{eq}^2 = \left(\frac{\omega}{c_c} \right)^2 - k_m^2$$

$$c_c = c, \quad \rho_c = \rho$$

2.6.2 Sound Hard Boundary (Wall) 1

EQUATIONS

$$-\mathbf{n} \cdot \left(-\frac{1}{\rho_c}(\nabla p_t - \mathbf{q}_d) \right) = 0$$

2.6.3 Plane Wave Radiation 1

EQUATIONS

$$-\mathbf{n} \cdot \left(-\frac{1}{\rho_c}(\nabla p_t - \mathbf{q}_d) \right) + i\frac{k_{eq}}{\rho_c}p + \frac{i}{2k_{eq}\rho_c}\Delta_{||}p = Q_i$$

2.6.4 Sound Soft Boundary 1

EQUATIONS

$$p_t = 0$$

2.7 PARTICLE TRACING FOR FLUID FLOW

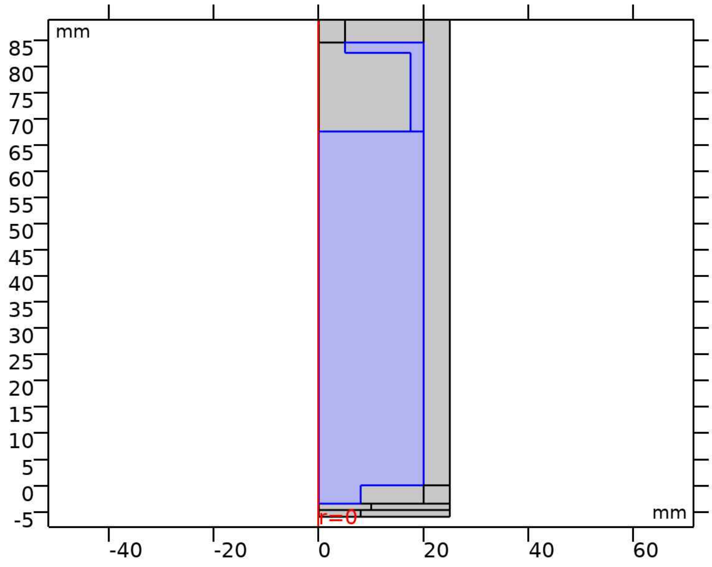

Particle Tracing for Fluid Flow

EQUATIONS

$$\frac{d(m_p\mathbf{v})}{dt} = \mathbf{F}_t$$

FEATURES

Axial Symmetry 1

Wall 1
Particle Properties 1
Release 1
Acoustophoretic Radiation Force 1
Drag Force 1
Gravity Force 1

2.7.1 Axial Symmetry 1

EQUATIONS

$$v_t = |\mathbf{v}_c| \sin\theta$$
$$v_n = |\mathbf{v}_c| \cos\theta$$
$$\theta \in \left[-\frac{\pi}{2}, \frac{\pi}{2}\right] \quad f(\theta) = \frac{1}{2}\cos\theta$$

where $\mathbf{v}_c$ is the particle velocity when striking the wall

2.7.2 Wall 1

EQUATIONS

$$\mathbf{q} = \mathrm{NaN}$$

2.7.3 Particle Properties 1

EQUATIONS

$$\frac{d(m_p\mathbf{v})}{dt} = \mathbf{F}_t$$

2.7.4 Release 1

EQUATIONS

$$\mathbf{q} = \mathbf{q}_0$$
$$\mathbf{v} = \mathbf{v}_0$$

2.7.5 Acoustophoretic Radiation Force 1

EQUATIONS

$$\mathbf{F}_{\mathrm{rad}} = -2\pi r_p^3\left[\frac{1}{3}\kappa_s \mathrm{Re}\left(f_0^{\mathrm{sl}} p^* \nabla p\right) - \frac{1}{2}\rho \mathrm{Re}\left(f_1^{\mathrm{sl}} \mathbf{u}^* \cdot \nabla \mathbf{u}\right)\right]$$
$$f_0^{\mathrm{sl}} = 1 - \tilde{\kappa}_s$$
$$f_1^{\mathrm{sl}} = \frac{2(\tilde{\rho} - 1)}{2\tilde{\rho} + 1}$$
$$\tilde{\kappa}_s = \frac{\kappa_{s,p}}{\kappa_s} \quad \tilde{\rho} = \frac{\rho_p}{\rho}$$

$$\kappa_s = \frac{1}{\rho c^2} \quad \kappa_{s,p} = \frac{1}{\rho_p \left(c_{p,p}^2 - \frac{4}{3} c_{s,p}^2 \right)}$$

2.7.6 Drag Force 1

EQUATIONS

$$\mathbf{F}_D = \frac{1}{\tau_p} m_p (\mathbf{u} - \mathbf{v})$$

$$\tau_p = \frac{\rho_p d_p^2}{18\mu}$$

2.7.7 Gravity Force 1

EQUATIONS

$$\mathbf{F}_g = m_p \mathbf{g} \frac{\rho_p - \rho}{\rho_p}$$

2.8 MULTIPHYSICS

2.8.1 Piezoelectric effect 1

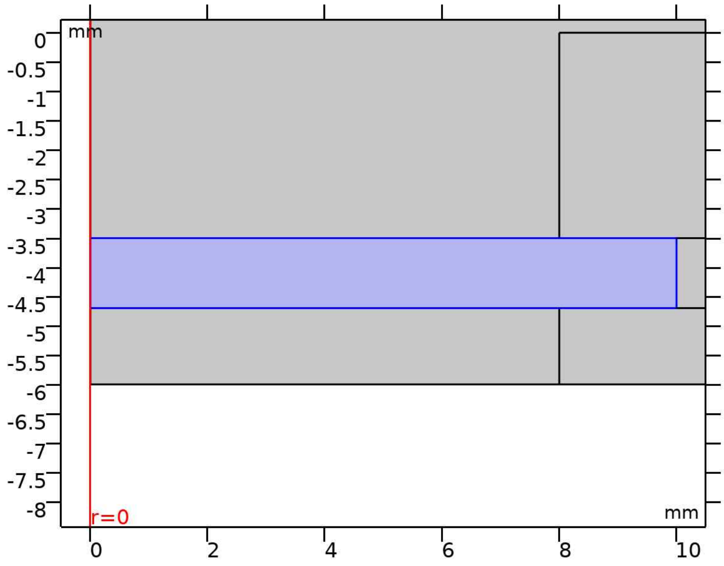

Piezoelectric effect 1

2.8.2 Acoustic-Structure Boundary 1

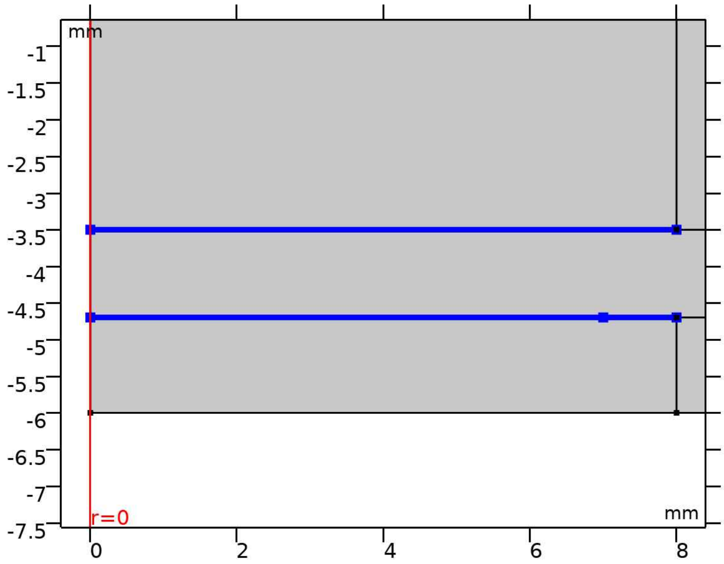

Acoustic-Structure Boundary 1

EQUATIONS

Exterior:

$$-\mathbf{n}\cdot\left(-\frac{1}{\rho_c}(\nabla p_t - \mathbf{q}_d)\right) = -\mathbf{n}\cdot\mathbf{u}_{tt}$$

$$\mathbf{F}_A = p_t\mathbf{n}$$

Interior:

$$-\mathbf{n}\cdot\left(-\frac{1}{\rho_c}(\nabla p_t - \mathbf{q}_d)\right)_{up} = -\mathbf{n}\cdot\boldsymbol{u}_{tt}$$

$$-\mathbf{n}\cdot\left(-\frac{1}{\rho_c}(\nabla p_t - \mathbf{q}_d)\right)_{down} = -\mathbf{n}\cdot\boldsymbol{u}_{tt}$$

$$\mathbf{F}_A = p_{tdown}\mathbf{n} - p_{tup}\mathbf{n}$$

2.9 MESH 1

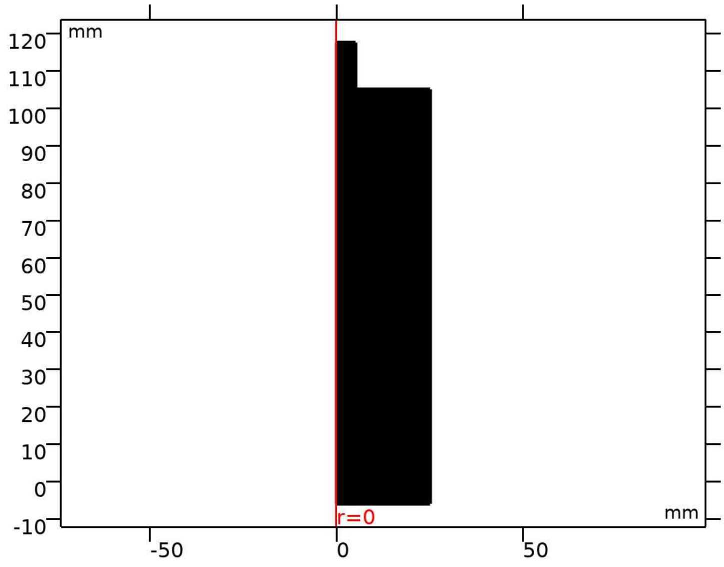

Mesh 1

3 Study 1

COMPUTATION INFORMATION

Computation time	22 s
CPU	Intel64 Family 6 Model 142 Stepping 9, 2 cores
Operating system	Windows 10

3.1 FREQUENCY DOMAIN

Frequencies (Hz)
f0

STUDY SETTINGS

Description	Value
Include geometric nonlinearity	Off

SETTINGS

Description	Value
Frequencies	1596000

PHYSICS AND VARIABLES SELECTION

Physics interface	Discretization
Solid Mechanics (solid)	physics
Electrostatics (es)	physics
Pressure Acaustics, Frequency Domian (acpr)	physics

MESH SELECTION

Geometry	Mesh
Geometry 1 (geom1)	mesh1

4 Study 2

COMPUTATION INFORMATION

Computation time	1 h 38 min 43 s
CPU	Intel64 Family 6 Model 142 Stepping 9, 2 cores
Operating system	Windows 10

4.1 TIME DEPENDENT

Times	Unit
range(0,0.001,0.03)	s

STUDY SETTINGS

Description	Value
Include geometric nonlinearity	Off

STUDY SETTINGS

Description	Value
Times	{0, 0.001, 0.002, 0.003, 0.004, 0.005, 0.006, 0.007, 0.008, 0.009000000000000001, 0.01, 0.011, 0.012, 0.013000000000000001, 0.014, 0.015, 0.016, 0.017, 0.018000000000000002, 0.019, 0.02, 0.021, 0.022, 0.023, 0.024, 0.025, 0.026000000000000002, 0.027, 0.028, 0.029, 0.03}

VALUES OF DEPENDENT VARIABLES

Description	Value
Settings	User controlled
Method	Solution
Study	Study 1

PHYSICS AND VARIABLES SELECTION

Physics interface	Discretization
Particle Tracing for Fluid Flow (fpt)	physics

MESH SELECTION

Geometry	Mesh
Geometry 1 (geom1)	mesh1

5 Result

5.1 DATA SET

5.1.1 Study 1/Parametric Solutions 2

SOLUTION

Description	Value
Solution	Parametric Solutions 2
Component	Geometry 1

5.1.2 Study 1/Solution 1

SOLUTION

Description	Value
Solution	Solution 1
Component	Save Point Geometry 1

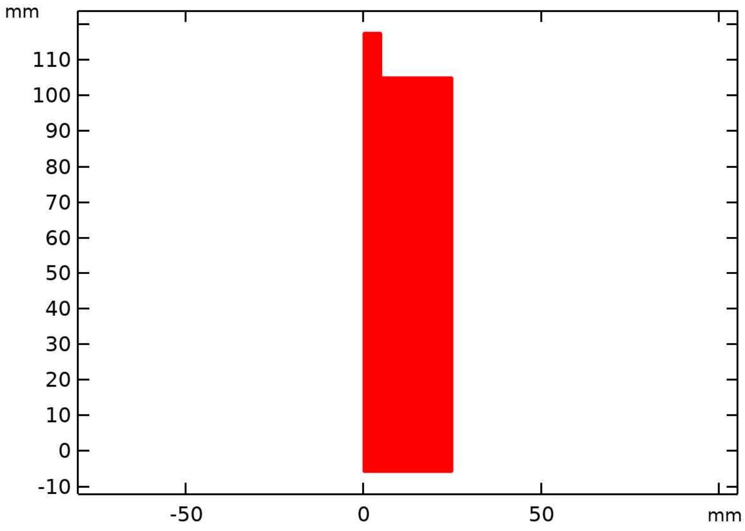

Dataset: Study 1/Solution 1

5.1.3 Revolution 2D 1

DATA

Description	Value
Dataset	Study 1/Parametric Solutions 2

AXIS DATA

Description	Value
Axis entry method	Two points
Points	{{0, 0}, {0, 1}}

REVOLUTION LAYERS

Description	Value
Start angle	-90
Revolution angle	225

ADVANCED

Description	Value
Define variables	On
Space variables	{rev1x, rev1y, rev1z}

5.1.4 Revolution 2D 2

DATA

Description	Value
Dataset	Study 1/Parametric Solutions 2

AXIS DATA

Description	Value
Axis entry method	Two points
Points	{{0, 0}, {0, 1}}

5.1.5 Revolution 2D 3

DATA

Description	Value
Dataset	Study 1/Parametric Solutions 2

AXIS DATA

Description	Value
Axis entry method	Two points
Points	{{0, 0}, {0, 1}}

REVOLUTION LAYERS

Description	Value
Start angle	-90
Revolution angle	225

5.1.6 Revolution 2D 4

DATA

Description	Value
Dataset	Study 1/Solution 1

AXIS DATA

Description	Value
Axis entry method	Two points
Points	{{0, 0}, {0, 1}}

REVOLUTION LAYERS

Description	Value
Start angle	-90
Revolution angle	225

ADVANCED

Description	Value
Define variables	On
Space variables	{rev4x, rev4y, rev4z}

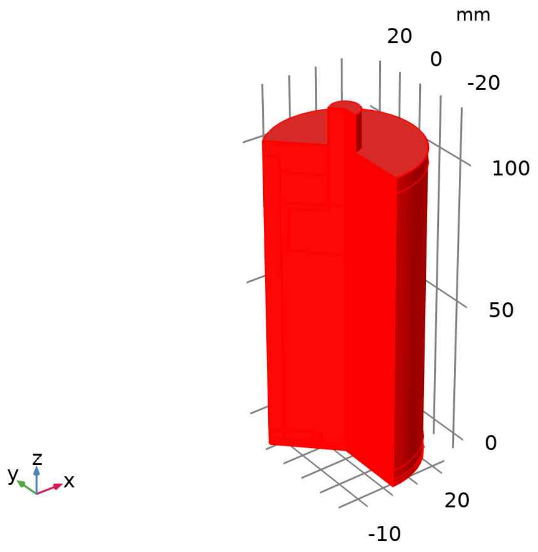

Dataset: Revolution 2D 4

5.1.7 Revolution 2D 5

DATA

Description	Value

Description	Value
Dataset	Study 1/Solution 1

AXIS DATA

Description	Value
Axis entry method	Two points
Points	{{0, 0}, {0, 1}}

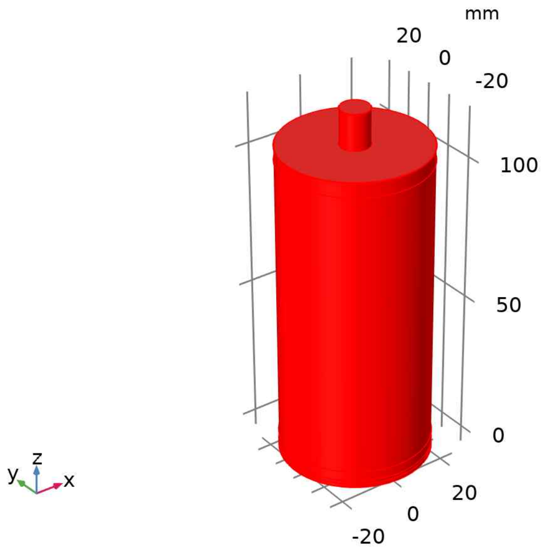

Dataset: Revolution 2D 5

5.1.8 Revolution 2D 6

DATA

Description	Value
Dataset	Study 1/Solution 1

AXIS DATA

Description	Value
Axis entry method	Two points
Points	{{0, 0}, {0, 1}}

REVOLUTION LAYERS

Description	Value
Start angle	-90
Revolution angle	225

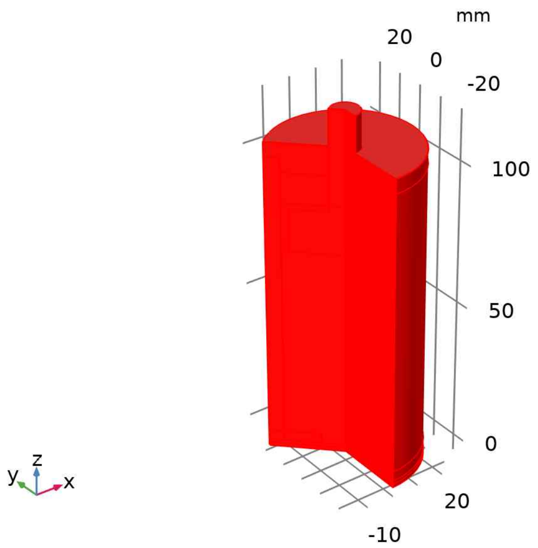

Dataset: Revolution 2D 6

5.1.9 Study 1/Parametric Solutions 3

SOLUTION

Description	Value
Solution	Parametric Solutions 3
Component	Geometry 1

5.1.10 Revolution 2D 7

DATA

Description	Value
Dataset	Study 1/Parametric Solutions 3

AXIS DATA

Description	Value
Axis entry method	Two points
Points	{{0, 0}, {0, 1}}

REVOLUTION LAYERS

Description	Value
Start angle	-90
Revolution angle	225

ADVANCED

Description	Value

Description	Value
Define variables	On
Space variables	{rev7x, rev7y, rev7z}

5.1.11 Revolution 2D 8

DATA

Description	Value
Dataset	Study 1/Parametric Solutions 3

AXIS DATA

Description	Value
Axis entry method	Two points
Points	{{0, 0}, {0, 1}}

5.1.12 Revolution 2D 9

DATA

Description	Value
Dataset	Study 1/Parametric Solutions 3

AXIS DATA

Description	Value
Axis entry method	Two points
Points	{{0, 0}, {0, 1}}

REVOLUTION LAYERS

Description	Value
Start angle	-90
Revolution angle	225

5.1.13 Study 2/Solution 77

SOLUTION

Description	Value
Solution	Solution 77
Component	Save Point Geometry 1

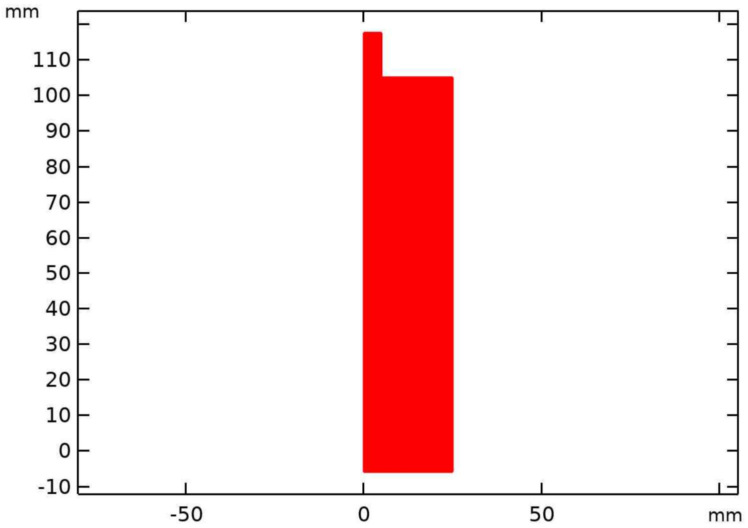

Dataset: Study 2/Solution 77

5.1.14 Particle 1

PARTICLE SOLUTION

Description	Value
Solution	Solution 77
Particle geometry specification	From physics interface
Physics interface	Particle Tracing for Fluid Flow

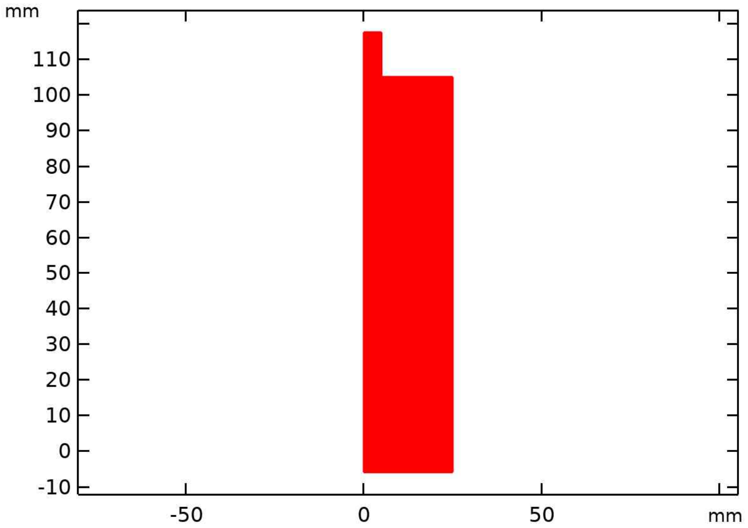

Dataset: Particle 1

5.2 TABLE

5.2.1 Evaluation 3D

Interactive 3D values

5.2.2 Evaluation 2D

Interactive 2D values

5.3 PLOT GROUPS

5.3.1 Stress (solid)

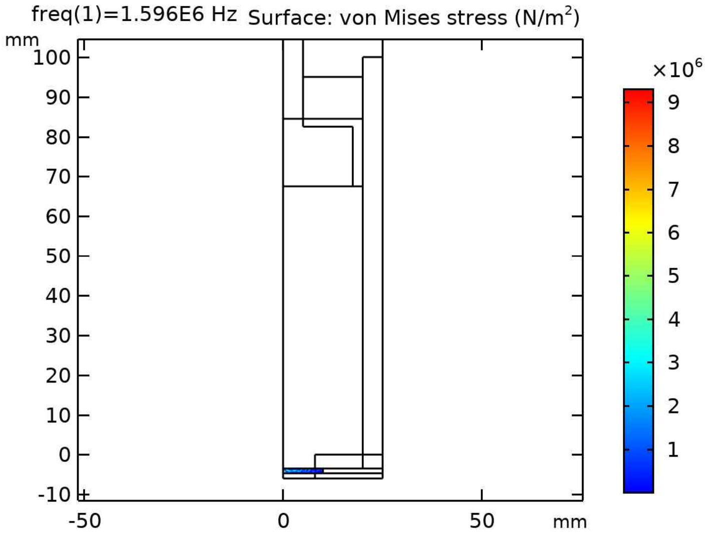

Surface: von Mises stress (N/m^2)

5.3.2 Stress, 3D (solid)

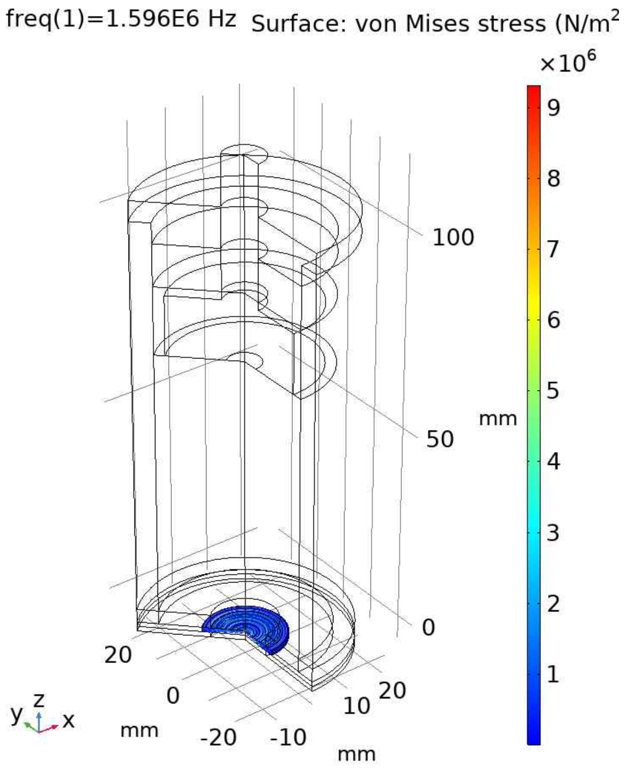

Surface: von Mises stress (N/m^2)

5.3.3 Electric Potential (es)

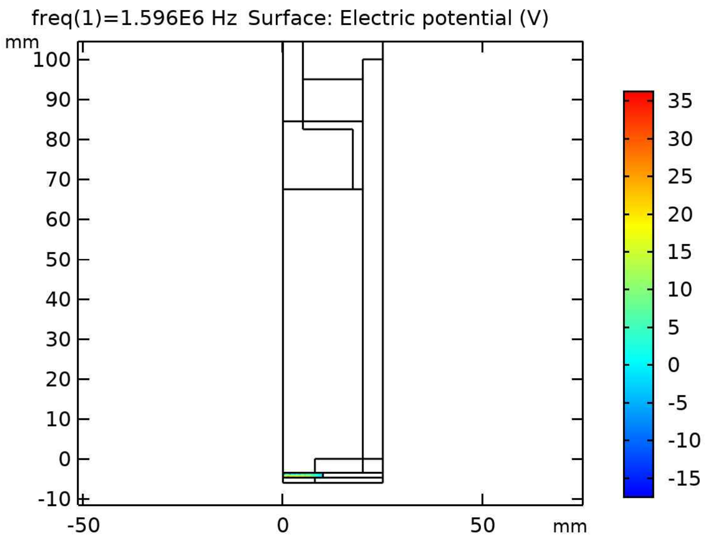

Surface: Electric potential (V)

5.3.4 Electric Potential, Revolved Geometry (es)

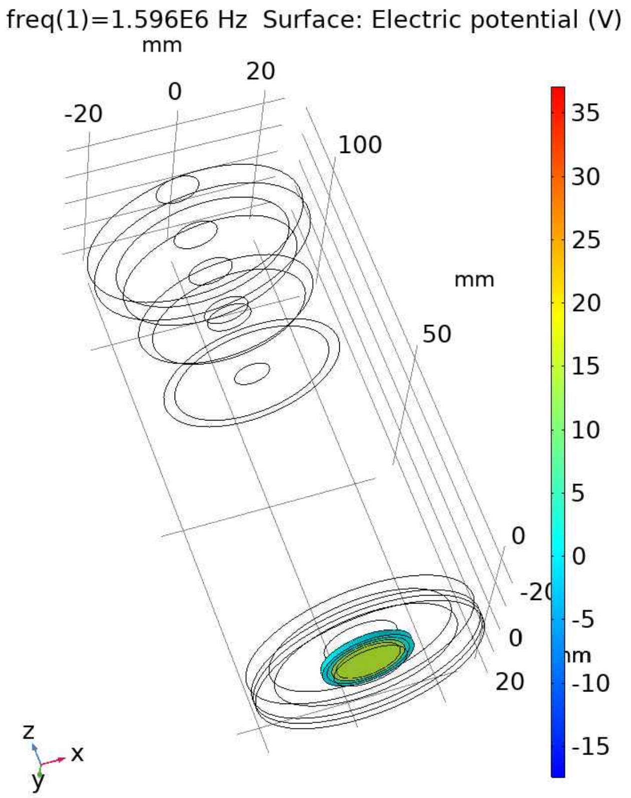

Surface: Electric potential (V)

5.3.5 Acoustic Pressure (acpr)

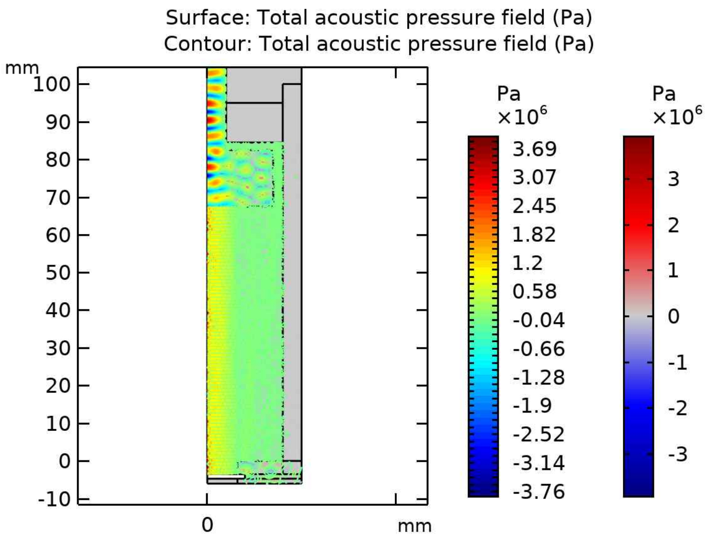

Surface: Total acoustic pressure field (Pa) Contour: Total acoustic pressure field (Pa)

5.3.6 Sound Pressure Level (acpr)

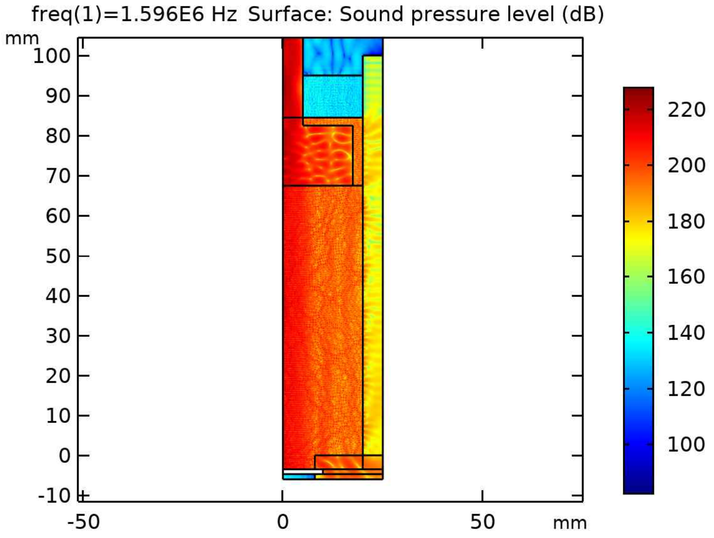

Surface: Sound pressure level (dB)

5.3.7 Acoustic Pressure, 3D (acpr)

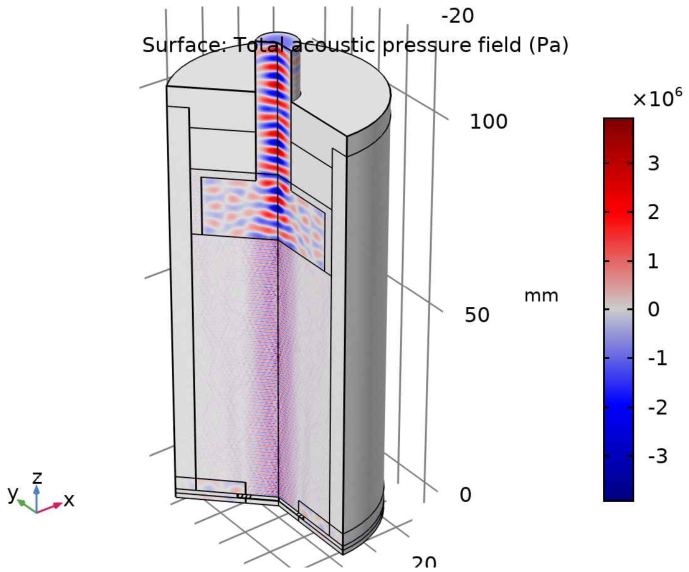

Surface: Total acoustic pressure field (Pa)

5.3.8 Sound Pressure Level, 3D (acpr)

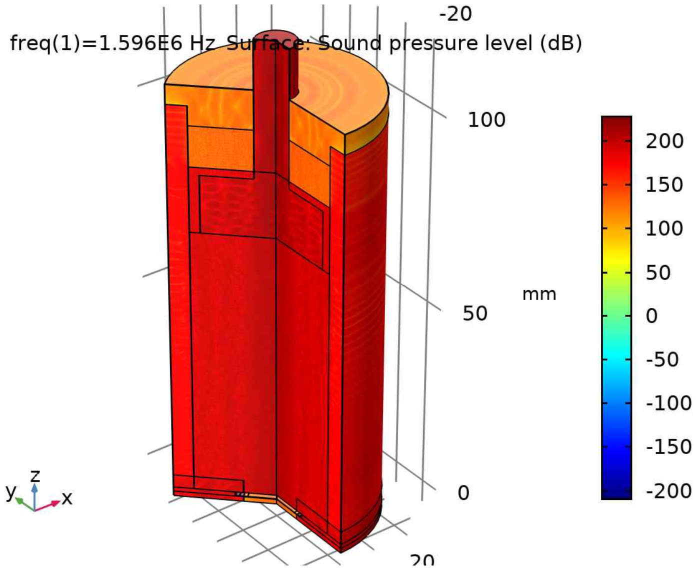

Surface: Sound pressure level (dB)

5.3.9 Particle Trajectories (fpt)

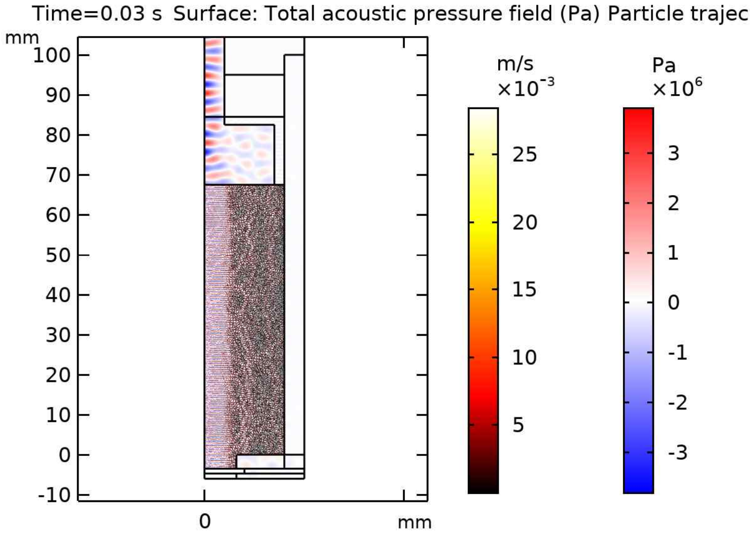

Surface: Total acoustic pressure field (Pa) Particle trajectories
